# Coexistence of balance and hierarchies: An ego perspective to explain empirical networks

**DOI:** 10.1093/pnasnexus/pgaf130

**Published:** 2025-04-29

**Authors:** Piotr J Górski, Adam Sulik, Georges Andres, Giacomo Vaccario, Janusz A Hołyst

**Affiliations:** Faculty of Physics, Warsaw University of Technology, Koszykowa 75, Warsaw 00-662, Poland; Faculty of Physics, Warsaw University of Technology, Koszykowa 75, Warsaw 00-662, Poland; Chair of System Design, ETH Zürich, Weinbergstrasse 56/58, Zürich 8092, Switzerland; Chair of System Design, ETH Zürich, Weinbergstrasse 56/58, Zürich 8092, Switzerland; Faculty of Physics, Warsaw University of Technology, Koszykowa 75, Warsaw 00-662, Poland

**Keywords:** signed networks, structural balance, status, hierarchy, data-driven agent-based modeling

## Abstract

The formation of positive and negative relations between individuals in social networks can be described by different approaches. Two prominent mechanisms are structural balance and status hierarchies. Balance motivates stability among friends and enemies in triads (e.g. an enemy of my friend is my enemy). Status considers respect and disregard originating from social hierarchy (e.g. positive relations towards those we respect). We demonstrate that integrating the two mechanisms through the concept of ego dynamics is key to understanding observable patterns in many social groups. We propose an agent-based model where dynamical changes result from agents aiming to resolve inconsistencies with structural balance and status. In contrast to previous models, our approach employs the ego perspective. Agents have limited, local knowledge and can only change their own relations. By fitting the model to real-world networks, we successfully replicated the observed over- and under-representations of certain triads in 36 empirical signed networks. This close matching to empirical data is achievable only by taking the ego perspective and not assuming global knowledge. Additionally, the model reveals that, when the status mechanism dominates, people in real networks tend to strive for the top of the hierarchy. Finally, our numerical simulations and analytic solutions demonstrate that a previously thought as continuous phase transition towards the paradise state (all links positive) can become discontinuous when the status mechanism is involved. This discontinuity indicates that desirable social configurations may, in fact, be quite fragile.

**Significance Statement**

Positive and negative social relations are subject to a tension between structural balance (e.g. “a friend of my friend is my friend”) and status hierarchies (e.g. “positive relations towards those of higher status”). We integrate both mechanisms by considering the ego perspective of an individual agent who can change only her own relations to others and possesses only limited information about others’ relations. In doing so, our model explains the observed over-representation of hierarchical triads across 36 empirical signed networks. Finally, analytically solving our model reveals that introducing status hierarchies renders desirable social configurations more fragile than when only structural balance dynamics is at play.

## Introduction

Signed relations are integral to social life and express individuals’ positive and negative sentiments towards each other. Individuals are aware of their relations to their immediate neighbors and also perceive the patterns of relations in their wider surroundings (1). Based on this information, individuals may change their relations. This evolution results in critical societal phenomena, such as consensus, polarization, and segregation. However, in data, e.g. voting for or against someone, declaring trust or distrust, recommending or warning against someone, one may learn about the signed relations individuals establish but not the mechanisms that generated them. This insight immediately raises the question of how to identify plausible mechanisms responsible for what is observed.

Structural balance (SBT) and status theory (ST) are among the most prominent theories explaining the evolution of signed relations (2, 3). They both rely on the same concept of cognitive dissonance minimization in signed triads (4). When individuals perceive contradictory information in their surroundings, they are pushed to action and adapt their signed relationships. Despite this shared foundation, social networks are typically modeled using SBT and ST processes separately, without accounting for their mutual influence (see, for example, (3, 5)). Our work bridges this gap by integrating these two perspectives into a unified framework of ego dynamics where an agent has limited, local knowledge and can only change her own relations to be consistent with either SBT or ST rules.

In SBT, a positive relation represents friendship and a negative one enmity. Individuals build balanced triads according to the following rules: “a friend of my friend is my friend,” “a friend of my enemy is my enemy,” “an enemy of my friend is my enemy,” and “an enemy of my enemy is my friend” (4, 6). Triads that do not respect these rules are considered unstable and are expected to change. For example, when a married couple experiences a difficult divorce, the resulting negative relation between them may lead mutual friends to take sides and alter their relationship with one of the divorcees. Local changes driven by SBT may have global implications pushing social organizations possibly through phase transitions to paradise (all links are positive) or polarized states (two hostile groups emerge) (7–10).

In ST, on the other hand, signed relations have directionality, and positive (negative) relations go from individuals of lower (higher) status to individuals with higher (lower) status (5, 11, 12). Then, dissonance arises from the absence of hierarchical structures, i.e. from inconsistent rankings of individuals according to their perceived status. For example, in a high school setting, when a student considered “popular” shows sympathy or deference for another schoolmate often bullied, it may lead other students to experience cognitive dissonance and then either quit bullying the victimized peer or prompt a change in attitude towards the “popular” student. This process creates local hierarchies that shape social organizations by defining power structures and promote stability (13–17).

Although positive and negative relations have different meanings in SBT and ST, they could or even should be modeled together. We often observe signed relations in the real world but not the underlying generative process. For example, an individual may establish a positive relationship with another individual because of shared friends, i.e. to construct a triad balanced according to SBT, or because of perceived status, i.e. to construct a triad hierarchical from her perspective. Although both processes can affect each other (16), a combination of them is rarely considered. Usually, they are treated separately, for example, to explain group formation and bullying among students (11, 18, 19) or predict signed links in online social networks (5, 20, 21). However, it is challenging to distinguish between SBT and ST in generating the observed relations, especially in social organizations that are not formally organized.

To provide a generative mechanism for the interplay of SBT and ST, we propose an agent-based model (ABM). Agents represent individuals, and their relations are mapped to signed links $G_{ij}$ in a directed signed network G. In contrast to previous works (7, 8, 22–24), we take an *ego perspective* (25). This leads to two important assumptions. First, each agent considers *only ego-triads*, i.e. triads composed of two links starting from the focal agent and a third link connecting her two neighbors. In such a triad, an agent relies on minimal external information—specifically, information about relations that do not directly involve the agent. Ego-based triads minimize external information requirements, as only knowledge of the single link connecting the two neighbors is needed. Hence, modeling ego-based triads is in line with the assumption that it is costly for agents to acquire external information. Second, agents may change only one of *their own* links when belonging to an unstable triad. The instability stems from the cognitive dissonance experienced by the focal agent and understood either through SBT or ST. To quantify the relative agents’ propensity of being influenced by ST over SBT, we introduce a control parameter *q*. With this ego perspective, we explicitly model cognitive dissonance at the agent level and explore how agents, through resolving it, may alter the network of relations. Thus, our model complements works considering triads, and not individual agents, as the focal entities driving the evolution of the network (7, 8, 22, 24). Such an approach aligns with (25), which postulates the importance of ego-based decisions in individuals’ perception of balance. By relying solely on local information, we analyze ego-based triads independently, creating a uniform dynamic framework for all ego-based triads without introducing additional parameters for different structure types. This ensures the parsimony of our model.

Our results show that the interplay of SBT and ST may either hamper or enhance the systems’ capability to reach a state where all signed links are positive (paradise state). Moreover, depending on the control parameter *q*, the model exhibits continuous and discontinuous phase transitions between disordered and paradise states. The discontinuous phase transition is a surprising characteristic not observed in the previous triad-focused SBT models (7, 8). This observation implies that thanks to the interplay between these two cognitive processes, a system far from a paradise state can still reach this state after changing very few relations. All these results are obtained from analytical calculations and are supported by numerical simulations that match remarkably well. Finally, we compare signed triad densities generated from the proposed model with empirical ones computed using three large-scale online communities and 33 relationship networks among high-school students. We find that our approach reproduces stylized facts observed in the real world, thus also bringing evidence of the empirical relevance of the modeled interplay. This matching to empirical data is achievable only by modeling the individual agents’ perspectives, highlighting the importance of the ego-based approach in agent-based models. Furthermore, we analyze additional agent and link characteristics (e.g. strong and weak signed relations) and identify significant correlations between these and the fitted model parameters. This enables broader conclusions about the underlying generative mechanisms and the factors influencing them.

In summary, our work makes several contributions. First, we integrate structural balance and status mechanisms into a unified ego-centric agent-based framework (based on the principle of minimal external information processed), addressing a much neglected gap in the study of signed relations. Second, by analytically solving the model, we uncover a surprising discontinuous phase transition to the paradise state, challenging traditional assumptions about the predictability of social configurations. Third, we complement our analytical results with robust empirical validation by reproducing patterns across 36 real-world networks, demonstrating the applicability and relevance of our model.

## Results

### Agent-based model for the evolution of ego-based triads

There are *N* agents and their relations are captured through a directed signed network G. For the analytical solutions presented hereafter, G is assumed to be a complete graph with N(N−1) directed signed edges, whereas in real-world case studies, we analyze other network structures. The edge structure remains fixed, but signs may change over time. Consequently, a pair of agents can have at most two distinct edges (one in each direction). The signs are considered as additional internal variables of every link. At each time step, an agent *A* is sampled uniformly at random and may change one outgoing link considering an *ego-based triad*. An ego-based triad is constructed in the following way. We sample two neighbors of *A*, labeled as *B* and *C*, to each of which *A* has a link ($G_{AB}$ and $G_{AC}$). Then, we sample one of the two directed links connecting *B* and *C*, e.g. $G_{BC}$. This sampling reflects the process of *A* meeting *B* and learning about *B*’s relation towards *C*. By this, we obtain a triad with three agents (*A*, *B*, *C*) and three directed signed links ($G_{AB}$, $G_{AC}$, $G_{BC}$). Then, if the triad is *unstable* (see below), the focal agent changes one of its outgoing links. Formally, this triad is a network subgraph, but not an induced subgraph (where all links of the network between the nodes *A*, *B*, and *C* are taken into account). This means that there can be other connections between the chosen nodes that are, however, not considered in a given time step. The great majority of such edges is then considered in another time step when a different ego-based triad, perhaps even consisting of the same three nodes, is constructed (see Materials and methods).

**Fig. 1.**
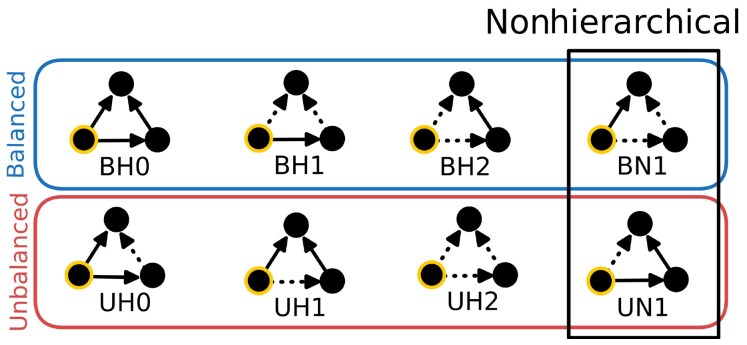
Ego-based triads. From an ego-based perspective, there are eight possible triads. An ego-based triad is constructed from the perspective of the focal agent (highlighted with a thick yellow border) and, hence, does not represent a subgraph with all existing network connections between the considered agents. Assuming agents possess a minimal amount of local knowledge, in the construction process, the focal agent learns the sign of one of the directed relations between two of her connected neighbors. Solid and dotted lines correspond to positive and negative links, respectively. When assessing the eight possible triads, four are structurally balanced (top) and four are unbalanced (bottom); six triads are hierarchical and two are nonhierarchical (in the box). Each triad is labeled using two letters and a number, **XY#**: X indicates whether the triad is structurally **B**alanced/**U**nbalanced, Y indicates whether the triad is **H**ierarchical/**N**onhierarchical, and # how many negative links are attached to the focal agent (**0**, **1**, or **2**). The balanced triads respect the rules: “friend of my friend is my friend,” “friend of my enemy is my enemy,” “enemy of my friend is my enemy,” and “enemy of my enemy is my friend.” Triads are hierarchical when a consistent ego-hierarchy (i.e. a hierarchy from the focal agent’s perspective) can be built. For instance, in the triad $\Delta_{BH0}$ with three positive links, the focal agent *A* respects *B* and *C* (symbolically as A<B,C) and knows that *B* respects *C* (i.e. B<C). Thus, $\Delta_{BH0}$ creates the consistent ranking A<B<C. For triads the $\Delta_{BN1}$ and $\Delta_{UN1}$, it is impossible to create a consistent ranking.

In total, there are eight possible ego-based triads, the stability of which depends on the cognitive dissonance that the focal agent *A* may experience according to either SBT or ST. We visualize these triads and their stability in Fig. 1 (see also Ref. (26)). According to ST, the six triads on the left in Fig. 1 are hierarchical and the two on the right are nonhierarchical. According to SBT, the four triads with zero or two negative edges are balanced, and the four triads with one or three negative edges are unbalanced.

Note that there are triads that are stable according to one theory but not the other. For instance, $\Delta_{BN1}$ is stable according to SBT and unstable according to ST. In our approach to model ST, explicitly defining the status of agents through an internal variable is not necessary. In other words, the hierarchy agents consider is not related to their internal attributes. It is instead based on the directed signed relations that represent the perception of agents. These perceptions may be passed to others, allowing agents to discern ego-based triads.

**Fig. 2.**
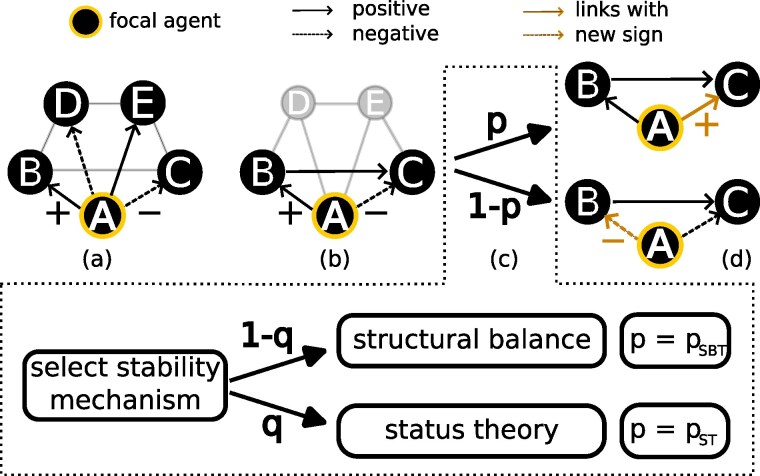
Model dynamics. There are four steps: a) pick a focal agent, b) construct a triad around the focal agent, c) select the mechanism for evaluating the triad stability: structural balance or status theory. Finally, d) if stable, keep the signs; otherwise, update the sign of one link. The probability *P* is the probability that a negative link becomes positive. This probability plays a role only when the chosen triad is unstable, and the outgoing links of the focal agent have varying polarities. The probability *P* is equal to $p_{\mathrm{SBT}}$ or $p_{\mathrm{ST}}$ in the case of structural balance or status, respectively. Note that the initial network (a) is just an illustrative figure that does not show the whole network with all directed edges.

With probability *q*, the focal agent evaluates the stability of the triads according to ST and with probability (1−q) according to SBT. Introducing the global parameter *q* allows us to model the interplay between SBT and ST. When the triad is stable according to the chosen theory, nothing happens. If the triad is unstable, the focal agent changes one sign of its outgoing links. For triads, in which both links of the focal agent are of the same sign, either one of them is chosen. If the links are of different signs, the negative link is chosen with probability $p_{\mathrm{SBT}}$ or $p_{\mathrm{ST}}$ when SBT or ST is chosen, respectively. Note that the global parameters $p_{\mathrm{SBT}}$ and $p_{\mathrm{ST}}$ may be different. The former describes the preference of agents for forming positive relations, whereas the latter—preference for respecting others. Besides this difference in interpretation, there are some similarities. They model the propensity to change a negative relation to a positive one, which relates to conflict avoidance. See Fig. 2 for a visual representation of one model step.

### Enhancing and hampering the accessibility to paradise

Based on the above rules, the signed network evolves and may reach absorbing states where there are only stable triads. When considering the triads’ stability according to SBT, such a network is composed of either (i) two polarized communities with all links within the communities being positive and all edges connecting the groups being negative or (ii) a *paradise* state with all links being positive. When considering ST, there are at least three different signed networks without nonhierarchical triads: a structure of agents respecting all other agents (i.e. all links positive/paradise), a structure of agents perceiving all others below themselves (i.e. all links negative), and a hierarchical network, where agents or groups of agents can be ranked from higher to lower status.

The signed network does not always reach an absorbing stationary state with only stable triads. It may also reach a *quasistationary state* where signs still evolve, but the density of positive links *ρ* fluctuates around a constant level. This level can be obtained analytically using the principle of detailed balance. In a quasistationary state, on average, the rate of changing links from positive to negative is equal to the rate of changing negative links to positive. Full formulas for the rates are given in the Supporting Information (SI). This dynamical balance condition can be reached when ρ=1. Hence, paradise is always a possible solution, which reflects that the paradise state is an absorbing stationary state under both SBT and ST. Besides the paradise state, there is also a second, nontrivial solution for the density of positive links *ρ* that can be derived from the equation:


(1) $$[2(1-q)(2p_{\mathrm{SBT}}-1)]\rho^{2}-[2(1-q)+(1-2p_{\mathrm{ST}})q]\rho +(1-q)=0.$$

**Fig. 3.**
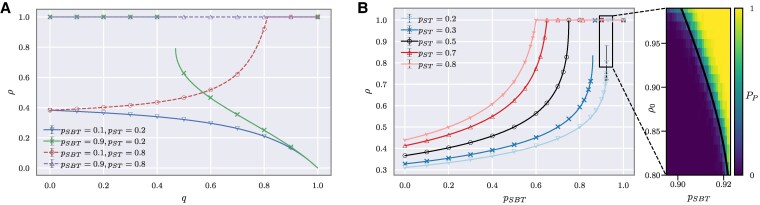
Continuous and discontinuous phase transitions between quasistationary and paradise states. The density of positive links *ρ* in the stable solution as a function of either (A) probability of choosing status over balance *q* or (B) preference for forming friendly relations $p_{\mathrm{SBT}}$. Analytical (lines) and numerical agent-based simulation (markers) results match perfectly. Depending on the preference for respecting others $p_{\mathrm{ST}}$, the system exhibits either a continuous (when $p_{\mathrm{ST}}>0.5$) or discontinuous (when $p_{\mathrm{ST}}<0.5$) phase transition. For the relation in (A), the transition is not observed when either both preferences ($p_{\mathrm{SBT}}$ and $p_{\mathrm{ST}}$) are large or both are small. (B) was obtained for equal importance of ST and SBT (i.e. q=0.5). The change of respect preference $p_{\mathrm{ST}}$ alters the critical point both for continuous and discontinuous cases. The inset on the right side highlights that with the jump, an unstable separatrix solution (the black curve) appears, which depending on the initial density $\rho_{0}$ differentiates basins of attraction of quasistationary and paradise states. Colors in the inset range from yellow (light) to purple (dark) to indicate the probability of reaching paradise state $P_{P}\in [0,1]$. Simulations were obtained for a complete graph of 100 agents. Each data point is the outcome of averaging simulations resulting from at least 100 different random initial conditions. A higher number of initial conditions were simulated for cases where standard errors were large in the ensemble of 100 simulation repetitions. Error bars are not shown because they are smaller than the marker size with the exception of the proximity of the critical point in (B).

Solutions of Eq. 1 for given values of parameters (*q*, $p_{\mathrm{SBT}}$, $p_{\mathrm{ST}}$) are, in general, the quasistationary values of *ρ*. These analytical values perfectly match numerical simulations of the agent-based model as shown in Fig. 3. For specific parameter values, ρ=1 can also be the solution of Eq. 1; below, it is discussed more thoroughly.

Before explaining the interplay of SBT and ST, we consider the limit case with q=0 in Eq. 1 and find that there is a continuous phase transition to the paradise state at $p_{\mathrm{SBT}}^{*}=0.75$. That means that, when agents evaluate triads exclusively based on SBT, the networks will have only positive links $\forall p_{\mathrm{SBT}}>p_{\mathrm{SBT}}^{*}$. Assuming structural balance dynamics only, a triad-focused perspective where any links in the triad may change at every time step was analyzed in Ref. (7). By contrast, in our agent-focused approach, agents can change only *their outgoing* links. In both perspectives, a similar phase transition to the paradise state is observed. However, for the triad-focused model, it occurs at the critical point $p_{\mathrm{SBT}}^{*}=0.5$, which means that, in our case, reaching the paradise state requires a much larger preference for forming positive relations. This difference is due to the fact that an unbalanced triad $\Delta_{UH0}$ in the agent-focused dynamics will surely flip its positive link, whereas with a triad-focused perspective, this would only happen with probability $\left(1-p_{\mathrm{SBT}}\right)$.

The density *ρ* changes monotonically with the control parameter *q* for fixed values of the parameters $\left(p_{\mathrm{SBT}},p_{\mathrm{ST}}\right)$ (see Fig. 3A). Hence, dependent on those parameters, increasing the probability *q* can either always increase or decrease *ρ* (assuming the increase in *ρ* is still possible, i.e. not in the paradise state). The former occurs when the preference for respecting others is high, $p_{\mathrm{ST}}>0.5$, and the latter when $p_{\mathrm{ST}}<0.5$. For the special case of $p_{\mathrm{ST}}=0.5$, the density *ρ* is independent of the probability *q* of choosing status over balance. Note that these results are not trivial as they hold no matter the value of the preference $p_{\mathrm{SBT}}$ for forming friendships in unbalanced triads. To understand this, let us look at Fig. 3A and consider the blue curve for which $p_{\mathrm{ST}}=0.2$ and $p_{\mathrm{SBT}}=0.1$. Here, we see that even when $p_{\mathrm{ST}}>p_{\mathrm{SBT}}$, increasing *q* still decreases the density of positive links *ρ*. That means that even though it is more likely to change a positive link according to ST than SBT, using ST more often decreases the number of positive links.

This puzzling phenomenon results from the different triads considered to be unstable following the two theories. According to ST, triads $\Delta_{UN1}$ and $\Delta_{BN1}$, respectively having one or two negative links, are unstable. The update that follows is affected by $p_{\mathrm{ST}}$. On the other hand, the process related to SBT considers the triads with one or three negative links as unstable and changes those. The parameter $p_{\mathrm{SBT}}$ does not influence all the changes. In particular, the triad with three negative links $\Delta_{UH2}$ is always converted to a new triad with two negative links, independently of the $p_{\mathrm{SBT}}$ value. This implies that when increasing *q*, triads $\Delta_{UH2}$ are rarely updated, which increases the density of negative links in the system.

### From continuous to discontinuous phase transitions

A phase transition between quasistationary (ρ<1) and paradise states (ρ=1) can be observed when taking one of the model parameters as the control parameter. Figure 3A and B respectively show this transition when using the parameters for the relative status importance *q* and for the preference for forming friendships $p_{\mathrm{SBT}}$ as control parameters (the case with $p_{\mathrm{ST}}$ is presented in the SI, Fig. S4). Precisely, the change of the control parameter may lead to (i) a continuous phase transition, (ii) a discontinuous one, or to no phase transition at all while keeping the system always in a (iii) disordered state, or in the (iv) paradise. All four cases are shown in Fig. 3A (also in SI, Fig. S3): (i) when $p_{\mathrm{SBT}}<0.75$ and $p_{\mathrm{ST}}>0.5$, (ii) when $p_{\mathrm{SBT}}>0.75$ and $p_{\mathrm{ST}}<0.5$, (iii) when $p_{\mathrm{SBT}}<0.75$ and $p_{\mathrm{ST}}<0.5$, and (iv) when $p_{\mathrm{SBT}}>0.75$ and $p_{\mathrm{ST}}>0.5$.

The continuous and the discontinuous phase transitions can also be observed in Fig. 3B. For the latter case, we also highlight the separatrix dividing the basin of attraction to the paradise and the quasistationary states (see the inset of Fig. 3B). When initialized with a density of positive links $\rho_{0}$ below the separatrix, the network reaches a quasistationary state (ρ<1). Otherwise, it reaches the paradise state (ρ=1). The analytical derivation of the critical values for the phase transitions and for the separatrix are presented in the SI, Section 2.

Mathematically, the observed phase transition in terms of the density *ρ* can be described as a catastrophic bifurcation, specifically a fold catastrophe (27, 28). In such a scenario, there are three solutions: two stable and one unstable. As the control parameter changes, at the phase transition point, the unstable and one of the stable solutions merge and disappear (i.e. they become imaginary). In our case, additionally, the control parameters and the density are constrained to realistic values, which limits the observation of typical catastrophe solutions to specific parameter regions.

The presence of discontinuous phase transition indicates that even though a signed network is very close to a stable quasistationary state, it might still reach the paradise state. That means an almost stable state with $\rho_{0}<1$ may evolve and contain only positive signed relations. This process occurs when $\rho_{0}$ is above a threshold value defined by the separatrix. Otherwise, the signed network reaches a quasistationary state with the density of positive links smaller than $\rho_{0}$. These phenomena are caused by the interplay of ST and SBT and are not observed when considering these theories separately.

### Under- and over-representation of ego-based triads

**Fig. 4.**
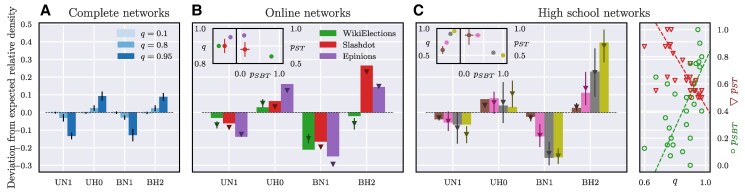
Under- and over-representation of ego-based triads in synthetic and real-world data. Panels present the deviation from the relative densities expected by SBT alone. (A) Complete networks. From SBT, one expects the relative densities of triads to be 1/3. When increasing *q*, the probability of choosing status over balance, the deviation from this value increases: nonhierarchical triads are under-represented, while hierarchical triads become over-represented. Other ABM parameters are $p_{\mathrm{SBT}}=p_{\mathrm{ST}}=0.5$. Similar deviations are observed in analyzed online social (B) or high school networks (C). Deviation panels (B and C) show values from data (bars) and from ABM simulations (corresponding markers) performed on respective topologies. The fitted values of ABM parameters are shown in the insets. As the high school dataset contains 33 networks, deviation bars were presented only for the subset of them. Obtaining larger deviations requires the model to have higher *q*. For the online networks, especially the nonhierarchical triads ($\Delta_{BN1}$) are extremely under-represented. For the high schools, varying levels of under- and over-representations were observed, but they were usually more profound for the balanced triads. Parameters of almost all schools are shown on the right part of (C), with dashed lines indicating correlations. Respect preference $p_{\mathrm{ST}}$ is highly anticorrelated with *q* (−0.90) and with friendship preference $p_{\mathrm{SBT}}$ (not shown here, −0.80), whereas *q* is correlated with $p_{\mathrm{SBT}}$ (0.67).

The interplay between SBT and ST changes not only the macrostructure of the network (e.g. *ρ*) but also its meso-structure. We indeed observe significant changes in the distribution of ego-based triads. When the dynamics is solely governed by SBT (q=0), triads with the same number of positive links are expected to have the same abundance. For instance, $\Delta_{BH1}$, $\Delta_{BH2}$, and $\Delta_{BN1}$ each constitute approximately 1/3 of all triads with one positive link (see Figs. S5 and S6 in SI). When increasing *q*, their abundances reveal instead significant differences as shown in the left panel of Fig. 4. We observe that $\Delta_{BN1}$ becomes *less* abundant compared to $\Delta_{BH2}$. Moreover, $\Delta_{UH0}$ becomes *more* abundant compared to $\Delta_{UN1}$. These changes occur as the nonhierarchical triads are converted into hierarchical ones by the dynamics induced by ST with the following conversion rates: $\Delta_{BN1}\overset{qp_{\mathrm{ST}}}{\to }\;\Delta_{UH0}$ and $\Delta_{BN1}\overset{q(1-p_{\mathrm{ST}})}{\to }\;\Delta_{UH2}$, or $\Delta_{UN1}\overset{q(1-p_{\mathrm{ST}})}{\to }\;\Delta_{BH2}$ and $\Delta_{UN1}\overset{qp_{\mathrm{ST}}}{\to }\;\Delta_{BH0}$. Triads $\Delta_{UH2}$ and $\Delta_{BH0}$ contain 3 or 0 negative links, respectively. They cannot be compared to other similar triads, so, further, we do not focus on them.

For the hierarchical triads, we observe that the numbers of $\Delta_{BH1}$ and $\Delta_{UH1}$ stay on the expected level, while the numbers of $\Delta_{BH2}$ and $\Delta_{UH0}$ vary dependent on the preference for respective others $p_{\mathrm{ST}}$ (see Figs. S7–S9 in SI). This phenomenon occurs as $\Delta_{BH1}$ and $\Delta_{UH1}$ cannot be created from the status dynamics defined with an ego perspective. This would not be the case when considering status dynamics defined at the triad level. In other words, the ego perspective creates an asymmetry in the relative abundance of triads with the same number of negative links. When observing relative abundances among the groups of triads with 1 or 2 negative links (as in Fig. 4), the deviations for those triads may be nonzero. This happens because the reduction of the nonhierarchical triads only possibly increases the abundance of hierarchical ones from all other groups. This is affected by $p_{\mathrm{ST}}$ (and also by $p_{\mathrm{SBT}}$), e.g. with $p_{\mathrm{ST}}=0$, transition$\Delta_{BN1}\overset{qp_{\mathrm{ST}}}{\to }\;\Delta_{UH0}$ will not happen. Summing up, nonhierarchical triads are always expected to be under-represented, while hierarchical triads, in the relative formulation, can be either under- or over-represented.

The over-representation of hierarchical triads is particularly interesting as it introduces an increase in the number of unbalanced triads, i.e. triads unstable according to SBT. Precisely, the nonhierarchical triad $\Delta_{BN1}$ may be converted into the hierarchical triad $\Delta_{UH0}$, which is unbalanced. Hence, in the final quasistationary state, there is a competition between the cognitive dissonance coming from SBT and ST. For increasing *q*, this competition decreases the number of nonhierarchical triads at the cost of elevating the number of unbalanced triads (see Fig. 4A).

### Traces of the SBT and ST competition

We now turn our attention to real-world data and check whether we find a similar under- and over-representation of triads as we would expect from our model. We analyze three large online communities (Epinions, Slashdot, and WikiElections datasets) and 33 school networks derived from the Spanish high school dataset. For all these, we have information about signed directed relations among different individuals that we use to reconstruct the different ego-triads and compute their statistics (see Materials and methods).

In the online communities, we find evidence for the competition between ST and SBT. In particular, nonhierarchical triads are significantly under-represented, while the unbalanced, hierarchical triads $\Delta_{UH0}$ are instead over-represented (see Fig. 4B or Fig. S12 in the SI). Moreover, the effect of ST seems to be the strongest for Epinions, which exhibits the lowest relative abundances of nonhierarchical triads. It is not clear whether ST is more pronounced in Slashdot or in WikiElections. The former has a lower relative abundance of $\Delta_{UN1}$, whereas the under-representation of $\Delta_{BN1}$ is larger for WikiElections.

Status influence is evidenced for the majority of school networks. Under-representation of nonhierarchical triads is observed for 32 networks in the case of $\Delta_{BN1}$, and for 28 in the case of $\Delta_{UN1}$ (see SI, Fig. S13). In Fig. 4C, we show the competition between status and balance for four school networks. There are networks with no or almost nonexistent deviations, but for some, they are large. For instance, among triads with two negative links, in the 4th reported network (dark gold), nonhierarchical $\Delta_{BN1}$ are under-represented with a deviation of −0.25, and deviation for over-represented $\Delta_{BH2}$ is 0.4. Hence (including the expected level of 1/3), the respective absolute abundances are 8% and 73%, which means that there are nine times more $\Delta_{BH2}$ than $\Delta_{BN1}$.

### Recovering the observed triad deviations

Via a parameter search, we fit our model to the 36 different datasets (see Materials and methods for details). Among the three online communities, we find that Epinions has the highest value of parameter *q* (see the inset of Fig. 4B), thus confirming the observation that this community has the strongest signature of ST. On the other hand, the fitting procedure on Slashdot and WikiElections results in *q* values compatible with each other. Hence, the ABM parameter does not provide evidence that any of those two networks have a higher level of status influence.

For the school networks, observed deviations are reflected in the fitted values of *q*. For the majority of schools, the status vs. balance parameter *q* varied between 0.6 and (almost) 1.0. Upon comparing values of different parameters across various school networks, we found a strong anticorrelation between preference for respective others $p_{\mathrm{ST}}$ and *q* (or $p_{\mathrm{ST}}$ and $p_{\mathrm{SBT}}$), see the right panel of Fig. 4C. This relation reflects a natural expectation that as the importance of hierarchy increases (higher *q*), individuals are more inclined to position themselves at the top of the hierarchy (lower $p_{\mathrm{ST}}$). Additionally, we note a mechanistic rationale underlying the relation between the propensities of creating a positive relation $p_{\mathrm{ST}}$ and $p_{\mathrm{SBT}}$: a decrease in $p_{\mathrm{ST}}$ necessitates an increase in $p_{\mathrm{SBT}}$ to maintain a consistent level of positive links.

### Relating the parameters to topological and individual properties

Further analysis of fitting the ABM to online networks reveals that in terms of $p_{\mathrm{SBT}}$ and $p_{\mathrm{ST}}$ values, Slashdot and Epinions are similar. For both of them, the preference for forming friendships $p_{\mathrm{SBT}}\approx 0$ and the preference for respective others $p_{\mathrm{ST}}$ is high, whereas for WikiElections $p_{\mathrm{SBT}}$ is high and $p_{\mathrm{ST}}$ is close to the intermediate level. These differences explain, among others, diverse deviations for $\Delta_{\mathrm{BH}2}$ (see also SI). For instance, with $p_{\mathrm{SBT}}=0$, triads $\Delta_{\mathrm{UN}1}$ and $\Delta_{\mathrm{UH}1}$ are always converted into $\Delta_{\mathrm{BH}2}$ through the SBT dynamics.

For the online networks, the close match in the obtained parameters for Slashdot and Epinions reflects that these networks originated from similar online interactions. Since in both communities, signs are related to assessing others’ articles (see Materials and methods), our results suggest that agents prefer to follow “the friend of my enemy is my enemy” and create a negative link. They are also more eager to respect others. When signs are related to voting (WikiElections), agents tend to form a positive link due to the balance dynamics.

Additionally, using linear regression analysis, we explore the relations between the parameter *q* and the characteristics of networks and students. The goal is to learn which of the characteristics influence the sign formation process identified here with the parameter *q*. The high school dataset provides additional information about, among others, signed connections’ strengths (weak/strong) and about individual students’ prosociality scores (see Materials and methods). We find that balance is more important when the network contains a higher density of stronger connections between students. To be more precise, densities of weak negative and weak positive links are significant explanatory variables with a positive influence on *q* as a dependent variable, whereas density of strong positive links has a significant negative impact. On the other hand, a supplementary exploratory analysis of the dataset indicates that strong links explain the variability of the density of balanced triads, but they do not explain hierarchical ones. We also find a weak but significant positive influence of the mean prosociality score on *q*. This means that a social group consisting of people with more frequent prosocial attitudes tends to shape this group according to social hierarchy. An exemplary linear regression model (P-value<0.001) with the density of weak negative links and mean prosociality score as independent variables explains 51% of *q* variability.

## Discussion

Our work contributes to understanding the evolution and structures of social organizations. The functioning of these organizations hinges on the underlying signed relations among their members (29–32). From the aggregation of these relations, a signed network emerges whose structure strongly influences the relations themselves and their stability. Structural balance theory commonly studies this influence, evaluating whether triads consisting of three individuals and their relations are balanced or not. However, this approach ignores the importance of status relations in social organizations. We have combined the two processes by acknowledging that both imbalance and a lack of hierarchy within triads may result in cognitive dissonance for a given agent, prompting her to adjust one of her signed relations (ego-based approach). Hence, we have created a unified generative mechanism that explains the evolution of signed networks and allows observation of nontrivial phenomena, including continuous and discontinuous phase transitions. Past analyses, including these theories separately, have already recognized the importance of such integration (3, 5). Here, we provide for the first time a trainable network agent-based model where the emergent network is the result of the two dynamics acting simultaneously.

The proposed generative mechanism helps to make sense of the mixed evidence about the impact of balance in signed networks reconstructed from real-world data (33). Were the signed networks purely the result of structural balance, the relative abundance of triads with a given number of negative links would be uniform. However, this is not the case for the majority of the analyzed data sets. Across the board, we observe that hierarchical triads are over-represented and nonhierarchical ones are under-represented. From our model, we can attribute this deviation from uniformity to the coexistence of balance and status. The two theories disagree on the stability conditions of triads, which leads to breaking the uniformity expected from structural balance alone. Our model recovers the deviations in the relative abundance of triads and defines a plausible mechanism for network formation.

To model balance and status together, we consider them as different manifestations of cognitive dissonance, requiring us to take an ego perspective. Previous work on structural balance primarily focused on triads rather than individuals for their update rules. This small difference has vast implications. First, from the conceptual point of view, it is now the agents themselves that decide to update *their* relations, not impersonal entities—triads. Moreover, agents base their decisions only on local information, which is essential when modeling social networks (26, 34). Second, this change in perspective implies a shift of 50% in the critical point of the model. Even without assuming status, the phase transition to the paradise state occurs at $p_{\mathrm{SBT}}^{*}=0.75$, instead of 0.5 expected from triad-focused dynamics (7). In social organizations, this implies that one does not need a slight preference to form positive relations but a large one. In other words, triad-focused dynamics underestimate how hard it is to achieve paradise assuming structural balance only. Third, only when taking the ego perspective, we can replicate over-representations observed in real social communities. Indeed, were we to assume the triad perspective, this close matching would disappear and be in disagreement with the data.

Note that our model consists of agents not possessing any additional features but still being able to perceive and compare relational patterns in their surroundings. As a consequence, using this formulation, we do not consider homophily, which is yet another prominent theory (3). Instead of looking at relations inside triads of agents, homophily takes into account pairwise interactions, which for some datasets may even suffice to reproduce the observed triadic (i.e. balance) statistics (35). However, using homophily requires agents’ meaningful attributes (gender, beliefs, etc.) to be known. Second, the obtained results neither explain nor model the emergence of social hierarchy, for example, originating from expectation states theory (14). We study how existing status influences the sign formation process. Combining status with structural balance resulted in having one network of relations of multiple natures. This makes the model helpful for analyzing signed datasets, for which the reasons how the relations formed are unknown or diverse. Moreover, different natures of connections are hypothesized to be the reason why theories, like status (12) or structural balance, are sometimes found questionable. That is why models that allow links of different meanings are essential to understanding the mechanisms of signed network formation.

Catastrophic behavior is a common characteristic of dynamic systems and is responsible for discontinuities (36, 37). This is also observed in the case of our model experiencing a fold catastrophe. Specifically, the ability of the system to reach paradise is heavily influenced by the interplay between status and structural balance. When agents are prone to respecting others ($p_{\mathrm{ST}}>0.5$), paradise is reached very often and in a continuous manner. Contrarily, when they tend to fight for the top of the hierarchy ($p_{\mathrm{ST}}<0.5$), paradise is harder to attain. Furthermore, it can only be attained through a discontinuous phase transition, which was not otherwise observed. Such transition implies a fragile paradise state. Despite this state being stable, small changes in individuals’ preferences may push the network far away from paradise and significantly impact the global structure when the separatrix between two domains of attraction is crossed.

The interplay between structural balance and status hence not only strongly influences expected structure in social organizations, but also their possible dynamics. Given the importance of this interplay, we have estimated it in various real-world networks by fitting the three model parameters (*q*, $p_{\mathrm{SBT}}$, $p_{\mathrm{ST}}$). Surprisingly, these parameters appear to be interrelated, with a particularly strong anticorrelation between *q* and $p_{\mathrm{ST}}$, indicating a tendency for people to aim for the top of the hierarchy when the hierarchy itself is more significant (see the right panel of Fig. 4C). Examining fitted parameters alongside available network and agent characteristics provides valuable insights into the signed links’ dynamics. Structural balance theory appears to have greater influence in social groups where there is a higher prevalence of strong relations among agents. Conversely, in networks with fewer strong relations, the significance of balance diminishes, magnifying the relative importance of status dynamics. The relationship between hierarchy and balance has been explored in previous work (38), where the status of agents (managers and subordinates) was predefined, different from the assumptions of informal relations in our model. Nonetheless, similar to our findings, higher levels of balance were observed for groups with a greater number of close relations. Furthermore, we found that social groups characterized by higher mean prosociality levels tend to establish stronger social hierarchies; such observation is consistent with findings suggesting that hierarchies facilitate cooperation (39).

When relating the model parameters to observed characteristics, caution must be exercised. Our analysis focuses on the observed cooccurrence of the phenomena without making definitive claims about causality, although the direct conclusions coming from applied methods might indicate that one observation explains the other one.

While acknowledging the above remark, one can still ask about the practical implications of the observed interdependencies. For instance, how can one bring a social organization closer to a paradise state where all relations are positive? A comparison of simulations on complete graphs and real-world networks, which are typically sparse, reveals that the network structure influences the expected density of positive relations: a larger expected value is obtained with a larger network density. Thus, in artificial networks adding more links will increase positive link density. On the other hand, in real-world social organizations, we can anticipate that the majority of newly added links are weak as individuals have limited capacities for creating and maintaining strong connections (40, 41). We have found that status importance increases in real social systems with a larger number of weak ties and is anticorrelated with the likelihood of individuals to form positive links. Hence, adding new (but weak) connections in real-world systems could actually be counterproductive compared to what one would assume simply by looking at simulations. In summary, in an artificial network without additional node or link features, a change in network density should be followed by an increase in positive link density. Such a simple outcome cannot be expected generally in real-world networks, where complexity arises when individuals and their relations possess additional characteristics. Indeed, based on our study, we instead expect the opposite. Specifically, considering reported trends such as the decline in the number of close friendships (42, 43) and a rise of acquaintances (44) in US society, we expect more negative relations in social networks when the networks are denser. Hence, instead of going closer to a paradise state with only positive relations, our findings suggest that the system may be trending towards a state of division and polarization.

Given our conclusion that systems, where structural balance is more influential, tend to have a higher density of strong relations, one could consider applying our results to the signed weight prediction problem (21). Indeed, some edge prediction methods (45) already incorporate SBT and ST. Our work may help in developing an algorithm that can dynamically scale the relative influence of balance and status in prediction by fitting the *q* parameter.

Although this paper focuses on human social networks, it does not differentiate between cases where signed relations are explicitly visible or inferred. In online social networks, relations are often more visible as they might be explicitly displayed, such as on user profile pages (46). In contrast, offline interpersonal networks might involve different perceptions of social relationships; for example, a student might conceal their feelings about another student or misinterpret acquaintances as friendship ties. The level of visibility introduces potential misinformation and errors in resolving cognitive dissonance within ego-triads. However, since only one signed relation in an ego-triad is susceptible to error, the results of the current model are unlikely to be significantly affected compared to scenarios where all perceived signs are prone to error. While limited visibility might slow down the convergence to final states and introduce noise around the separatrix between different system phases, no substantial changes in the overall dynamics are expected. Nevertheless, this aspect of visibility opens intriguing possibilities for extending the model to incorporate misinformation and cognitive errors, offering a deeper characterization of bounded rationality.

Potential extensions of our model could incorporate agents’ cognitive dissonance not only in ego-based triads but also in other motifs and network types. Like most studies in the SBT literature, we focus on triads. However, cycles of varying lengths may also contribute to cognitive dissonance. Building on SBT measures that assign different weights to cycles of varying lengths (47), we believe that such cycles may influence dynamics on different timescales. For the sake of parsimony, we refrain from including them. Additionally, the model could be extended to accommodate different types of networks, such as those with missing, neutral or weighted links (48), or multilinks. However, implementing such modifications requires access to data featuring these types of links while also ensuring overfitting is avoided.

Another promising direction of further research is to examine how ego perspectives and the combination of structural balance and status theories explain triad densities in other types of signed networks. Examples include organizational networks, such as international relations (49), or animal networks (50), where structural balance has demonstrated predictive power regarding relational changes. These networks are also likely influenced by status dynamics, making them suitable for the adaptation of our model. Investigating such networks could reveal distinct patterns in fitted parameters or their correlations across network types, providing deeper insights into the underlying dynamics.

In conclusion, we have introduced a generative model employing the ego perspective in signed networks, emphasizing individual agents rather than triads and their direct experience of cognitive dissonance. From the theoretical point of view, adopting this perspective significantly shifts the critical point of the transition between the unbalanced state and paradise. Our model demonstrates the possibility of a discontinuous phase transition, highlighting the inherent fragility of the system. In practice, the ego perspective enables testable predictions for real-world data. Our findings underscore that structural balance plays a more prominent role in social groups characterized by a higher density of strong relations, such as close friendships and rivalries, while status dynamics becomes more pronounced in groups with fewer strong ties. Additionally, our results point to a tendency for individuals to strive for the top of the hierarchy, and higher levels of prosociality in groups where status dynamics is more influential. Overall, our model provides a valuable benchmark for assessing the relative impact of structural balance and status dynamics in social organizations, enabling the reconciliation of previous conflicting findings regarding signed networks and the generation of new hypotheses.

## Materials and methods

### Signed directed networks

We considered signed, directed networks. The nodes are agents and the links are their relations. Precisely, agents have a positive relation with those they like (i.e. consider a friend) or respect (i.e. consider of having a higher status) and have a negative relation with those they dislike (i.e. consider an enemy) or disrespect (i.e. consider of having a lower status). In the model, agents do not possess any attributes.

### Network structure

We consider different types of network structures. The analytical results are obtained considering complete graphs. These are networks where every agent has directed links to all other agents. This implies that a network with *N* agents has N(N−1) directed links. The analytical results have been compared with numerical simulations. For phase transition analysis (see Fig. 3), we simulate a complete graph with N=100 agents. For the triad deviations’ analysis (see Fig. 4A), we simulate a complete graph with N=32 agents. When analyzing the empirical data, we use the observed network structures. Precisely, when fitting the model parameters to the datasets, we used the reported relations between individuals. The network structures emerging from the aggregation of relations are usually sparse, but with high clustering. For more details, see Data section below.

### Ego-based triads

An ego-based triad is defined by a set of three links between three agents. Two links go from a focal agent *A* to two other agents *B* and *C* and the third link is from *B* to *C*. At each time step, an ego-based triad is chosen and evaluated by the focal agent. The procedure to choose the ego-based triad depends on the network structure. In the case of complete graphs, we choose an ordered sequence of three agents sampled uniformly at random, e.g. (*A*, *B*, and *C*). Then, we consider the ego-based triad having *A* as the focal agent and the link from *B* to *C*. For networks with different structures, we first remove links not belonging to any triads. Then, we sample uniformly at random an agent *A* and one of *A*’s neighbors—*B*. Finally, from the set of common neighbors, a third and last agent *C* is chosen uniformly at random.

Note that ego-based triads are not all the possible directed triads that can be formed in a trio of agents. In theory, all connection patterns within a triad can be classified using the triad census metric (51), which identifies 16 possible patterns for unsigned networks. Among these, nine patterns involve a pair of unconnected agents. Such triads are excluded from our dynamics because the absence of a link implies the absence of a defined relationship and the knowledge of whether one agent (dis)likes or (dis)respects another. These disconnected triads cannot induce cognitive dissonance from a balance/status perspective and are thus irrelevant to the focal agent’s perception. Similarly, a pattern forming a triadic cycle is also not considered. This is because, for these triads, there is no agent with two outgoing links, as all agents have one outgoing link. In other words, in cycles, focal agents have too little or require more information in order to evaluate the triad’s stability (see also the next section on this matter). Six connection patterns from the triad census can be derived using one or more ego-based triads (see SI, Section 1). During each dynamical step, only one ego-based triad is evaluated, but other triads may be selected in subsequent steps.

Our approach of focusing on ego-based triads is similar to the measure proposed in (24), where only four out of six patterns constituted the network balance level. In that measure, the trio of agents was considered together with all the links, meaning that connection patterns were balanced if they consisted of only balanced specific ego-based triads. Differently from Ref. (24), we take the agent and not the triad perspective: a trio of agents may be stable from one agent’s point of view and, at the same time, unstable, taking a different agent as the focal agent.

Previous SBT agent-based models assumed triad-based dynamics, but in our study, the previous approach fails to capture heterogeneity in the densities of different hierarchical triads (as shown in SI, Fig. S10). The ego perspective is crucial not just because we analyze ego-based triads but because the need for sign changes originates from the experienced cognitive dissonance. As discussed also by Ref. (25), unbalanced triads that the agent is unaware of do not drive changes. Thus, the ego perspective is essential both theoretically and empirically, as it allowed us to fit the model and derive meaningful conclusions.

### Information asymmetry, bounded rationality, and cognitive dissonance

There is information asymmetry between the agents. Each agent is aware of its own signed relations towards the other agents. However, agents do not know what are the relations that the other agents have towards them. In other words, we assume that agents do not have global knowledge of the network, they instead have very limited local knowledge.

We assume that directed links are strictly related to the agent they go out from. This agent decides who she likes, respects, etc. In other words, outgoing links constitute agents’ internal knowledge. Agents know and remember with whom and what type of relations they formed in the past. To form triads and evaluate their stability, such internal information is not enough. Ego-based triads are triads that require the smallest amount of external information (i.e. information coming from other agents), see SI Table S1. For all other triad pattern types, including cyclic triads or fully induced subgraphs, the focal agent would require additional external information, which makes such triads more difficult to be constructed (they require more effort) and evaluated. Therefore, our dynamics considers the analyzed ego-based triads only. However, when assuming a low cost of external information, one can imagine extended ABM dynamics with all types of triads considered. This would possibly require additional model parameters, making it harder to fit those parameters and use the model.

We assume that agents are bounded rational and consider only one ego-based triad at a time. Bounded rationality of agents means that (i) agents rely on local information, a fundamental principle of social network models (34) and (ii) they have limited cognitive ability to process this information. From an ego perspective, information about triads being fully induced subgraphs can be considered local. However, in a fully connected network, evaluating them requires processing six pieces of information: two internal (the ego’s outgoing links) and four external (the remaining signed links). The bounded rationality helps determine the minimal information agents should retain. First, the agent keeps the two internal pieces, as these are already known to the ego and come at a lower cognitive cost. Next, the agent has to choose from the four external pieces. The two pieces connecting the ego’s neighbors define the ego-based triads analyzed in this study. The remaining two are discarded as they form semicycles of length 2, which are also associated with distinct social processes such as reciprocity and homophily. We do not model these processes. Incorporating shorter semicycles would require additional parameters (e.g. different update rates for semicycles of varying lengths, and separate relative status vs. balance influence parameter), complicating the model and making parameter calibration more challenging. Thus, assuming bounded rationality, a focal agent *A* considers only three links: two outgoing links to *B* and *C* and a third link coming from *B* to *C*. The information about the link between *B* and *C* is kept only during that specific time. Also, if *A* has *B* in multiple ego-based triads, this does not matter. The focal agent *A* considers only the ego-based triad established with *B* and *C*. Consequently, our model follows asynchronous dynamics (52), where each agent operates autonomously, and a randomly selected agent makes a decision at each step. We believe this approach better reflects real-world interactions compared to a scenario where the entire triad “meets” and makes a collective decision.

Focal agents may experience cognitive dissonance when ego-based triads are unstable. The stability is defined either considering status theory or structural balance (see Fig. 1). To resolve the dissonance, the focal agent changes the sign of one of its links in the triad.

When status is chosen to evaluate the stability and the triad is found unstable, the focal agent can either change the negative link to positive or the positive to negative. The former action is taken with probability $p_{\mathrm{ST}}$, and the latter with probability $\left(1-p_{\mathrm{ST}}\right)$. When structural balance is chosen, a triad is unbalanced if it contains either one or three negative links. If the links of the focal agent have the same sign, one of these links is changed. If one link is negative and the other is positive, they are chosen with probabilities of $p_{\mathrm{SBT}}$ and $\left(1-p_{\mathrm{SBT}}\right)$, respectively. The model algorithm is sketched out in Fig. 2.

### Initial density of positive links

Due to the principle of ergodicity, in complete networks, initial conditions are irrelevant unless the system is already balanced or in close proximity to a balanced state or to the separatrix. In most cases, initial density of positive links $\rho_{0}=0.5$ was applied. For other network structures, the system might not be ergodic, and $\rho_{0}$ does matter. For example, there may exist subgraphs that will always stay unbalanced, e.g. triad $\Delta_{UH1}$ with an additional negative link from *C* to *B*. When links $G_{BC}$ and $G_{CB}$ do not belong to other triads where either agent *B* or *C* is a focal agent, these links will never align. As a consequence, one of the triads (*ABC* or *ACB*) is always unbalanced. Creation of such triads is related to $\rho_{0}$. Therefore, for other network structures, varying initial densities of positive links ranging from 0 to 0.9 were used.

### Data

The three popular datasets often used in the study of signed networks, structural balance and status theories are Epinions, Slashdot, and WikiElections (5, 20, 53–56). Epinions dataset (N≈44 k, number of directed triads T≈11 M) contains, among others, trust-distrust links between users of a web page. Users gave their opinions in the form of reviews, which were further rated by other users. The Slashdot dataset (N≈27 k, T≈1.25 M) is also based on a web page. Slashdot gathers a community writing about science, technology, and politics. Users submit articles that are further reviewed by the community. Users may tag other users as *friends* or *foes*. WikiElections (N≈4 k, T≈745 k) is a dataset containing votes from elections for Wikipedia administrators. Each submission for an administrative function has to be voted on by users. Voters can vote *for* (+) or against (−). This results in a directed network with signs on links. Summing up, these datasets consist of human online interactions, and the observed signs may originate both from friendship/enemy or respect/disrespect relations. For online datasets, signed links may vary in accessibility. For instance, they are publicly available for Slashdot, were less straightforward to determine for WikiElections, and uncertain for Epinions as the website is no longer operational. In the described model, we assume that users evaluate a single triad without verifying multiple link signs simultaneously.

The Spanish high school dataset (57–59) encompasses 13 high schools giving information about signed links among students and providing students’ characteristics, such as prosociality score, cognitive reflection score, gender, grade and class. In 10 schools, there were no links between students from different grades. Thus, different grades were treated as separate networks, which allowed us to extract 33 separate networks ranging *N* = 26–534 and *T* = 1,015–143,112.

Additional network statistics related to the datasets are given in SI, Tables S2–S4.

### Fitting ABM parameters

To fit the model parameters to a given network, we simulated the ABM using the observed number of nodes and the observed network structure. Then, we performed a grid search for the parameters for which the simulated density of positive links in the quasistationary state and the corresponding deviations were closest to the observed ones. The smallest grid step was Δq=0.025, $\Delta p_{\mathrm{SBT}}=\Delta p_{\mathrm{ST}}=0.1$ for online networks, and Δq=0.025, $\Delta p_{\mathrm{SBT}}=\Delta p_{\mathrm{ST}}=0.05$ for high school dataset. To evaluate how well the model performed, we defined an error function to be the mean squared error between simulated and real deviations for the $\Delta_{1}$ and $\Delta_{2}$ triads. Parameter sets resulting in an error value smaller than 110% of the smallest error value were used to calculate the mean and standard deviation of fitted ($q,p_{\mathrm{SBT}},p_{\mathrm{ST}}$). The standard deviation and the grid step were further used to calculate the fitted parameters’ uncertainty. Fig. 4B presents the results of the fitting procedure for online networks. For detailed fitting results of specific high school networks, refer to Section 5 of the SI along with Figs. S14–S16, and Table S5.

### Exploring Spanish high school dataset

To explore the relationships between the dataset and the status vs. balance parameter *q*, we applied linear regression analysis with *q* as a dependent variable. Among the exploratory variables, we considered network characteristics, triangle network characteristics (a triangle network is a network where triads become nodes and triads sharing links are connected), signed network properties, and agent traits. The full list is given in the SI, Section 6A. We found that networks with large density in the triangle network are outliers, so we removed them from the analysis. There were six such networks, which were also the networks with the smallest number of agents; four of them consisted of students belonging to the same class. Using the remaining 27 networks, we applied linear regression with weights being the inverse squared root of *q* uncertainty.

We performed data exploration on the individual level (see SI, Section 6B). The goal was to verify whether the conclusions about the influence of strong/weak links’ density or mean prosociality score on the competition between balance and status could also be found from the data itself. For the linear model density of balanced triads as a function of densities of strong and positive links, both variables were found significant (with *P*-values below 0.001). On the other hand, for a similar model but with the density of hierarchical triads as a dependent variable, the density of strong links was not significant. This means that the observed influence of strong links is directly related to SBT, and the competition is affected only indirectly. We also studied correlations between prosociality scores with densities of balanced or hierarchical triads. Both correlations (0.14 and 0.10, respectively) were found significant. This means that in networks with higher average prosociality of agents, both balanced and hierarchical triads appear more often. The dataset exploration does not show how agents’ prosociality affects the competition between balance and status.

## Supplementary Material

pgaf130_Supplementary_Data

## Data Availability

Publicly available datasets were used in this research, with proper citations provided in the reference list. Section 4 of the SI includes dataset hyperlinks if the referenced papers do not contain the data directly. The codes used in this work are available: https://github.com/pjgorski/Balance-and-Hierarchy-ABM.
