# Supplementary material for: Coexistence of balance and hierarchies: An ego perspective to explain empirical networks
Source: PNAS Nexus. 2025 Apr 29;4(5):pgaf130. doi: 10.1093/pnasnexus/pgaf130 (PMC12080543; doi:10.1093/pnasnexus/pgaf130)
Supplement: pgaf130_Supplementary_Data [file pgaf130_supplementary_data.pdf]

PNAS nexus

Supporting Information for

Coexistence of balance and hierarchies: An ego perspective to explain empirical networks

Piotr J. Górski, Adam Sulik, Georges Andres, Giacomo Vaccario, and Janusz A. Hołyst

To whom correspondence should be addressed.
E-mail: piotr.gorski@pw.edu.pl

This PDF file includes:

Supporting text
Figs. S1 to S17
Tables S1 to S8
SI References

Supporting Information Text

1. Processing of external information for triads

In the proposed model, we consider the ego perspective. That is, triads are analyzed from the focal agent's point of view. In the dynamics procedure, we also assume that the amount of external information agents process is minimal. We distinguish two kinds of information: internal and external. Internal information is agents' outgoing signed connections. Agents by themselves decide on what type of relation they have with other agents: whether they like, dislike, respect or disrespect them. External information for a given agent is outgoing signed relations of other agents. These are opinions of other agents. In our model, we assume that internal information is the agents' intrinsic property, something they always possess and know about. On the other hand, they do not keep the knowledge about external pieces of information – this is something they need to find out anytime they try to evaluate triads' stability.

While constructing a triad for processing, the focal agent acquires certain pieces of external information. In the model, we assume that acquiring external information is costly. One could imagine that the focal agent considers varying groups of agents in which she is involved to assess the stability of these groups, which further can result in the agent's cognitive dissonance, leading the agent to alter one of her connections. In theory, the agent could analyze groups consisting of 2, 3, 4 or more agents. Following other agent-based models with structural balance dynamics, we assume that agents evaluate triads, that is, groups of three agents. Triadic census shows connection motifs that may appear between three nodes (1). For unsigned graphs, there are 16 distinct triadic motifs. 9 of them contain pairs of agents without a relation. Such motifs are not of our interest because the focal agent cannot construct a triad to evaluate. The rest of them are shown in Fig. S1. Considering that different agents from those seven triads could be focal agents, in Tab. S1, we present the number of external pieces of information that the focal agent needs to process the triad. Agent A of motif 030T requires one piece of external information, which is the least amount for all motifs, for all possible focal agents. Our agent-based model dynamics assumes that agents are able to acquire one piece of information, and therefore the only possible subgraph they are able to process is the motif 030T. Such subgraphs we call ego-based triads. Fig. 1 of the main paper shows all signed motifs that may be the outcome of the triad construction process.

The fact that in the dynamics, focal agents may construct and process only the subgraphs resembling the motif 030T does not mean that all trios of agents with more links (than in 030T) are rejected and not considered during the simulation. In fact, most of the remaining seven motifs (Fig. S1) can be constructed and processed using a number of ego-based triads. For instance, 120U contains two distinct ego-based triads; see Fig. S2: one with A as the focal agent and the other with B as the focal agent. A motif 120U is not processed as a whole, but sequentially during different simulation steps. It can be shown that motifs 030T, 120D, 120U, 210 and 300 can be derived using ego-based triads. The motif 030C cannot be derived and is not considered by the dynamics. The motif 120C is considered by the dynamics, excluding the edge $(B \to A)$ that is never involved in the stability evaluation.

2. Analytical solution for agent-based model

A. Notation and assumptions. The way of obtaining analytical equations is the extension of methods applied in Ref. (2) where Local Triad Dynamics (LTD) model was introduced.

In the derivation below, we use the following notation:

1. Model parameters:
   - q – probability of choosing status dynamics in the update step,
   - p_{SBT} – probability of changing a negative link to a positive one when an unbalanced triad (Δ_{UH1} or Δ_{UN1}) is picked when SBT dynamics was chosen,
   - p_{ST} – probability of changing a negative link to a positive one when a nonhierarchical triad (Δ_{BN1} or Δ_{UN1}) is picked when ST dynamics was chosen.
2. Variables important for deriving analytical formulas:
   - ρ – density of positive links,
   - n_k – density of triads with k positive links,
   - π^+ – rate of changing a positive link to become negative,
   - π^- – rate of changing a negative link to become positive.
3. Other variables:
   - q^*, p^*_{SBT}, p^*_{ST}, ρ^* – values of parameters in the phase transition point
   - $\tilde{q}^*$, $\tilde{p}^*_{SBT}$, $\tilde{p}^*_{ST}$, $\tilde{\rho}^*$ – values of parameters that would appear in the phase transition point without considering the realistic ranges.

Possible ranges of model parameters and the densities are $[0, 1]$. It is assumed that triads in each n_k group are indistinguishable. That means each of them can be randomly picked with the same probability. This assumption surely works for complete graphs. For incomplete networks, assuming an agent-based algorithm (i.e., starting with choosing an agent and not a whole triad), some triads can be picked more frequently than others.

It is further assumed that link signs are random (no correlations, no clusters, etc.). In such a case, the relation between ρ and n_k can be written as a binomial distribution:

$$n_k = \binom{3}{k}(1-\rho)^k \rho^{3-k}. \quad [1]$$

In directed networks, the groups n_1 and n_2 are degenerate; that is, there are 3 different triads with 1 negative link and 3 different triads with 2 negative links. One of the triad types is non-hierarchical in both of these groups. All other triads are hierarchical. In the analytics, we assume that the densities of different triad groups are equal. Although for large q in the agent-based model simulations, we observe over- and under-representations of triad densities, and thus this assumption becomes wrong, the analytics results still correctly predict the phase transition. As a consequence, our analytical results do not address over- and under-representation of triads. These we obtain numerically only.

B. Derivation. When the system is not in a paradise state, it reaches a *quasi-stationary state*. This state is not frozen, the signs still evolve but they fluctuate around the quasi-stationary levels. These levels can be obtained numerically using a time average.

The analytical result describing the quasi-stationary state is obtained using a detailed balance approach. In the quasi-stationary state, on average, the number of positive links that become negative is equal to the number of negative links that become positive. This can be written as an equality between transition rates:

$$\pi^+ = \pi^-. \quad [2]$$

Taking into account the knowledge about the agent-based dynamics, those rates can be written as:

$$\pi^+ = \frac{1}{3}q(n_1+n_2)(1-p_{ST}) + (1-q)n_1(1-\frac{2}{3}p_{SBT}) \quad [3]$$

$$\pi^- = \frac{1}{3}q(n_1+n_2)p_{ST} + (1-q)(\frac{2}{3}n_1 p_{SBT} + n_3) \quad [4]$$

Using the equality (2), one obtains that $\rho = 1$ is always a possible solution together with the solutions of the following quadratic equation [which is the Eq. (1) from the main paper]:

$$\Big[2(1-q)(2p_{SB}-1)\Big]\rho^2 - \Big[2(1-q) + (1-2p_H)q\Big]\rho + (1-q) = 0 \quad [5]$$

Thus, the set of all solutions comes from the 3rd-order algebraic equation, and the system experiences a fold catastrophe, which is described in the next section.

The equation (5) is the base of all analytical solutions for specific values of model parameters. For instance, one can easily check when the quasi-stationary state exists. The above solution comes from the equation of the form $a\rho^2 + b\rho + c = 0$. It follows the quasi-stationary state does not exist (i.e., the two solutions are in complex space) when $\Delta = b^2 - 4ac < 0$.

C. Relations between critical values. When $\Delta \geq 0$, the quasi-stationary state may exist (one still needs to check if the obtained ρ value(s) are in the realistic range $[0, 1]$). The fold catastrophe happens at the point where the two solutions of Eq. (5) become equal, that is, for $\Delta = 0$. The values of parameters in the phase transition are called critical and are denoted with $*$ superscript, e.g., p^*_{SBT}. By $\tilde{p}^*_{SBT}$, we will denote critical values of parameters without considering the realistic ranges. From $\Delta = 0$, one can easily obtain equations for $\tilde{\rho}^*(q, p_{ST})$ and $\tilde{p}^*_{SBT}(q, p_{ST})$.

$$\tilde{\rho}^* = \frac{2(1-q)}{2(1-q) + (1-2p_{ST})q} \quad [6]$$

$$\tilde{p}^*_{SBT} = \frac{1}{2}\left[\frac{\Big(2(1-q) + (1-2p_{ST})q\Big)^2}{8(1-q)^2} + 1\right] = 0.5 + \Big(0.5 + (1-2p_{ST})\frac{q}{4(1-q)}\Big)^2 \quad [7]$$

The above equations do not take into account the possible space of parameters. If the critical values are outside the range, then the phase transition is either not observed in the system or is continuous. From Eq. (6), one can see that the value of density of positive links in the transition point does not depend on the p_{SBT}. When $p_{ST} = 0.5$, then the critical density $\rho^* = \tilde{\rho}^* = 1$ no matter the value of q, and the transition is continuous. When $p_{ST} < 0.5$, then the critical density $\rho^* = \tilde{\rho}^* < 1$ is decreasing with the value of q, and the transition is discontinuous. When $p_{ST} > 0.5$, then $\tilde{\rho}^*$ is larger than 1. This is outside the realistic range, as the density cannot exceed 1. Thus, $\tilde{\rho}^* \neq \rho^*$, and the disappearance of the quasi-stationary state, i.e., the transition, appears earlier exactly for $\rho^* = 1$, and the transition is continuous. Using Eq. (5), one can calculate critical values

of parameters for which $\rho = 1$ is reached. If such values do not exist, then no quasi-stationary solution is reachable, and the only solution is the paradise state (see, for instance, the purple line in Fig. 3a in the main paper). Thus, Eq. (7) is valid, i.e., $\tilde{p}_{SBT} = p_{SBT}$, when $p_{ST} \leq 0.5$. Otherwise, using Eq. (5), we obtain $p^*_{SBT} = 0.75 - \frac{1}{4}\frac{q}{1-q}(2p_{ST} - 1)$. It means that for $p_{ST} > 0.5$, the dependence of p^*_{SBT} on p_{ST} is linear, whereas for $p_{ST} \leq 0.5$, it is quadratic.

Interestingly, Eqs. (6) and (7) have very similar form, which allows to write the relation between critical ρ and p_{SBT}:

$$p^*_{SBT} = 0.5 + \frac{1}{4(\rho^*)^2}. \qquad [8]$$

Equations for $\tilde{q}^*(p_{SBT}, p_{ST})$ and $\tilde{p}^*_{ST}(q, p_{SBT})$ are given below.

$$\tilde{q}^* = \frac{2\left(1 - 2\sqrt{p_{SBT} - 0.5}\right)}{1 + 2p_{ST} - 4\sqrt{p_{SBT} - 0.5}} \qquad [9]$$

$$\tilde{p}^*_{ST} = 0.5 - \frac{1-q}{q}\left(2\sqrt{p_{SBT} - 0.5} - 1\right) \qquad [10]$$

The above formulas show that the fold catastrophe values of parameters q and p_{ST} exist only when $p_{SBT} \geq 0.5$. For $p_{SBT} < 0.5$, no matter the values of q and p_{ST}, the two solutions of Eq. (5) are always real, and the catastrophe does not happen. The catastrophe can be observed in realistic parameter ranges when $p_{SBT} > 0.75$. Then, the transition is discontinuous. For $p_{SBT} = 0.75$, all three solutions converge in $\rho = 1$, and the transition is continuous. For $p_{SBT} < 0.75$, the catastrophe occurs outside realistic ranges, and thus if the transition exists, it is continuous and occurs in $\rho = 1$. One can calculate the transition points by taking Eq. (5) with $\rho = 1$.

Combining the above conclusions and the ones that follow Eqs. (6-7) give us more specific conditions for observing phase transition. With q as a control parameter, phase transition will be observed and will be discontinuous when $p_{SBT} > 0.75$ and $p_{ST} < 0.5$. The transition will be continuous when $p_{SBT} < 0.75$ and $p_{ST} > 0.5$. Otherwise, it won't be observed. This is shown in Fig. 3a in the main paper or in Fig. S3.

Summing up the above derivation, we obtain the following equations for the critical values of positive link density ρ and system parameters (q, p_{SBT}, p_{ST}). These equations contain conditions for p_{SBT} and p_{ST}. They do not contain conditions for q, as they do not have a compact form.

$$\rho^* = \begin{cases} \dfrac{2(1-q)}{2(1-q) + (1-2p_{ST})q} & \text{if } p_{ST} \leq 0.5 \\ 1 & \text{if } p_{ST} > 0.5 \end{cases} \qquad [11]$$

$$p^*_{SBT} = \begin{cases} 0.5 + \left(0.5 + (1-2p_{ST})\dfrac{q}{4(1-q)}\right)^2 & \text{if } p_{ST} \leq 0.5 \\ 0.75 - (2p_{ST} - 1)\dfrac{q}{4(1-q)} & \text{if } p_{ST} > 0.5 \end{cases} \qquad [12]$$

$$q^* = \begin{cases} \dfrac{2\left(1 - 2\sqrt{p_{SBT} - 0.5}\right)}{1 + 2p_{ST} - 4\sqrt{p_{SBT} - 0.5}} & \text{if } p_{SBT} > 0.75 \text{ and } p_{ST} < 0.5 \\ \dfrac{3 - 4p_{SBT}}{2p_{ST} + 2 - 4p_{SBT}} & \text{if } p_{SBT} < 0.75 \text{ and } p_{ST} > 0.5 \\ \varnothing & \text{otherwise} \end{cases} \qquad [13]$$

$$p^*_{ST} = \begin{cases} 0.5 - \dfrac{1-q}{q}\left(2\sqrt{p_{SBT} - 0.5} - 1\right) & \text{if } p_{SBT} > 0.75 \\ 0.5 + \dfrac{2(1-q)}{q}(0.75 - p_{SBT}) & \text{if } p_{SBT} < 0.75 \end{cases} \qquad [14]$$

D. Parameter p_{ST} as the control variable. Fig. S4 shows the phase transition in ρ when probability p_{ST} is the control variable. As was discussed in the previous subsection, probability p_{SBT} above or below the value of 0.75 is decisive for the observed type of phase transition.

E. Quasi-stationary solutions. Eq. (5) describes the quasi-stationary solutions. In general, there may be two of them. Both are observed in the case of a discontinuous phase transition. When the transition is continuous, one of them (the separatrix) is outside the realistic range of parameters.

3. Under- and over-representation of ego-based triads

A. Triad densities and deviations in complete networks. Without dynamics related to status theory, i.e., when q is close to 0, triads with the same number of positive links have approximately the same abundance. Triads Δ_{BH1}, Δ_{BH2} and Δ_{BN1} constitute 1/3 of all triads with one positive link, and triads Δ_{UH1}, Δ_{UH0} and Δ_{UN1} constitute 1/3 of all triads with two

positive links. This can be observed in the left bar for each group of triads in Fig. 4a in the main paper or in Fig. S5 and Fig. S6 here. Fig. S5 shows the true triad densities. Due to the difficult comparison of respective triads, in Fig. S6, we also show the deviations. These are the densities normalized among each group and further decreased by 1/3. Note the order of values on the Y-axis.

When the importance q is larger, for the hierarchical triads, the numbers of Δ_{BH1} and Δ_{UH1} stay on the expected level, while the numbers of Δ_{BH2} and Δ_{UH0} vary dependent on p_{ST}. This is shown in Figs. S7-S9. Figs. S7 and S8 show triad deviations for different values of parameters p_{SBT} and p_{ST}. The sizes of deviations are much larger than when the ST influence is small. To show whether the numbers of Δ_{BH1} and Δ_{UH1} truly stay on the expected level, we compare true triad densities to densities expected in the random model [following Eq. (1)], which is shown in Fig. S9.

When analyzing deviations, we see that the values for triads Δ_{BH1} and Δ_{UH1} are in most cases below the values for Δ_{BH2} and Δ_{UH0}. However, as shown for real systems, it can be the opposite (see Δ_{BH2} for WikiElections in Fig. 4b in the main text or Fig. S12 here). Such behavior is hardly visible for complete graph networks. One exception can be a region of low p_{ST} values for $q = 0.8$ in Fig. S7 where the deviation for Δ_{UH0} is smaller than the one for Δ_{UH1}. However, the model is able to reproduce varying deviations for real-world networks, which shows that such behavior may be the characteristics of the topology.

B. Ego perspective gives real-world deviations. Triad-focused dynamics. Varying deviations are possible due to the ego perspective. Fig. S10 shows the triad densities that one could obtain in the triad-focused model. In such a model, when an unstable triad is chosen, then any of its links may change. In the triad-focused model, there are no varying deviations, and the difference between densities of numerically observed ones and the expected values from Eq. (1) for the hierarchical triads is small. Thus, it is the ego perspective that allows us to obtain varying over-representations, as we show in Fig. 4 in the main text.

4. Additional statistics of datasets

All original references for these datasets were given in the main text. The exact sources of downloaded datasets are as follows: WikiElections – (3), Slashdot – https://snap.stanford.edu/data/soc-sign-Slashdot090221.html, Epinions – https://www.kaggle.com/datasets/masoud3/epinions-trust-network, high school networks – (4).

Tab. S2 shows the details about the online datasets. Tables S3 and S4 provide details about the 33 high school networks. In those networks, all agents participated in at least one ego-based triad. A limited number of edges that had not been part of any triads were removed. In total, there were 131 such edges, 83 of them in $t1$, $t2$ and $t6$ networks. In all online and school datasets, considered triads are subgraphs consisting of three agents and three links not forming loops (as in Fig. 1 in the main paper). Among each trio of agents, there can be 6 triads, at the maximum. As described in Section 1, all ego-based triads of a given trio of agents are considered by the dynamics not at a single but during different simulation steps. Cyclic triads are not considered. For the analyzed datasets, the density of cyclic triads constitutes, on average, 1.4% of all trios of agents with connections existing between each pair of agents, see Fig. S11.

A. Triangle graph definition. One of the differences between complete graph and real-world topologies is that in the former, all triads are interconnected, whereas in the latter, the triad connectivity is heterogeneous. This is important because, with high triad connectivity, each sign change randomly alters the stability of many triads (in the complete graph: $3(N-2)$. We suppose this leads to the creation of the quasi-stationary state and eventually to the phenomenon of phase transition in complete graph networks. Thus, networks with varying triad connectivity may behave differently, and one should not compare directly the obtained values of fitted network model parameters (q, p_{SBT}, p_{ST}).

To overcome this and measure how ego-based triads are connected, we created a triangle graph (TG) transformation. The definition follows the *line graph* definition. Our triangle network is a directed graph. Each node corresponds to one ego-based triad of the original network, so the number of nodes of the triangle graph equals the number of ego-based triads of the original network. Links in the triangle graph are defined as follows. Let us denote by T_i a generic i-th triad, and by Δ_{ABC} an ego-based triad with links having the following directions: $A \to B$, $B \to C$ and $A \to C$. A directed link connects a triad T_i to a triad T_j if the triad T_i may affect the triad's T_j stability.

With our agent-based dynamics, a triad Δ_{ABC} can affect the stability of a triad Δ_{ABD} but cannot affect the stability of a triad Δ_{BCE}, because instability in Δ_{ABC} cannot alter the sign of $B \to C$. On the other hand, both triads Δ_{ABD} and Δ_{BCE} may cause stability change of triad Δ_{ABC}. Therefore there exists bidirected links between triads Δ_{ABC} and Δ_{ABD}, and a single directed link from Δ_{BCE} towards Δ_{ABC}.

For high school networks, we constructed respective triangle graphs and calculated their connectivity (Tab. S3).

B. Under- and over-representation of ego-based triads in data. Deviations from the expected relative densities for online and high school networks are shown in Figs. S12 and S13.

5. Fitting agent-based model to high school dataset

Figs. S14-S16 compare deviations for the best fit of agent-based simulations with the true deviations for all high school networks. The deviations shown in the main paper are for the following school networks: $t11\_4\_1$, $t11\_6$ (both in Fig. S15, $t11\_9\_4$ (Fig. S14) and $t11\_2\_4$ (Fig. S16).

One can see that, overall, the ABM is able to reproduce deviations in many different circumstances. The most common case are over-representations for hierarchical Δ_{BH2} and Δ_{UHO}, under-representations for nonhierarchical Δ_{BN1} and Δ_{UN1}, and being between for Δ_{BH1} and Δ_{UH1}. Such cases are reproduced very well. However, there are cases with varying deviations for balanced and unbalanced triads. See, for instance, school network $t11\_9\_3$: the ABM best fit gave slightly too small deviations for Δ_{BH2} and too big for Δ_{UN1}. It is even possible (like for network $t11\_10\_3$) to have extremely big deviations for the balanced triads and lack of them for the unbalanced. The model is not designed to fit such nonhomogeneous behavior, and the best fit gives deviations between the true ones. To overcome this, one could extend the status mechanism in our model to treat differently unbalanced and balanced nonhierarchical triads. This would require including more parameters.

Tab. S5 shows fitted values of parameters and their uncertainties for different schools. Fitted values were obtained by taking an average of parameters that gave simulations with triad deviations closest to the real ones. As described in Materials and Methods in the main paper, it was performed as follows. The chosen error function was a mean squared error between simulation and real triad deviations. We identified the parameter set with the smallest error (and results for this parameter set are shown in Figs. S14-S16). Then, we found all parameter sets with error values not larger than 110% of the smallest value. From this set we took an average and standard deviation. The average is the fitted value. The uncertainty was calculated using the following equation: $\delta_x = \sqrt{\frac{(\Delta x)^2}{3} + (s(x))^2}$, where x can be one of (q, p_{SBT}, p_{ST}), $s(x)$ is the standard deviation from identified values from different sets, and Δx is the smallest grid step of the parameter in the ABM simulations.

6. Exploring high school dataset

A. Regression analysis. In the regression analysis of fitted q as a dependent variable, we used the following exploratory variables: all columns as shown in Tables S3 and S4, densities of undirected triads (n_0, n_1, n_2 and n_3), densities of students with specific values of prosociality and CRT scores. We tested the significance of all or subsets of variables, with the highest focus on agent traits (gender, prosociality, CRT scores) or link signs (densities of weakly/strongly positive/negative links), because these could have the clearest interpretation.

We applied simple linear regression and weighted linear regression with weights being the quadratic inverse of deviations of fitted q. The obtained results were similar. Below we describe the latter.

First, we found that the variable that explains datasets most is TG density. Fig. S17 shows the relation between q and TG density. We identify the six networks with large TG density and low q as outliers. Four of these networks are the only networks (out of 33) that consist of a single class of students (see Tab. S4). The other two are networks with the lowest number of agents and considerably lower number of triads than in all other networks. That is why we decided to remove these six networks from the regression analysis.

When repeating the analysis after this removal, triangle network density was not a significant exploratory variable of q. Tab. S6 shows significant linear regression models.

Before discussing Tab. S6, let us note that with this analysis, we do not imply causality. Variables density of weakly positive or negative links have a significant positive influence on q. The influence is negative in the case of the density of strongly positive links. Mean prosociality has a positive influence on q. The standard deviation of prosociality and the density of students with the lowest prosociality score (soc_0) are both anti-correlated with mean prosociality (correlation is equal to -0.79 and -0.68, respectively). Thus, in the models, those two metrics have a significant negative influence on q. We do not see a clear interpretation of the variability of prosociality. The result related to density soc_0 might mean that anti-social people have the greatest impact on shaping the whole group in terms of status-balance competition. The density of triads with 2 negative links n_2 has a positive influence on q, however we do not see a clear interpretation of this variable.

B. High school data exploration on the individual level. The main two conclusions coming from the regression analysis are (1) the higher density of weak links, and (2) the higher mean prosociality, the larger influence of status dynamics as compared to structural balance dynamics. To verify whether these conclusions can also be found in the data themselves, we correlated the density of balanced and density of hierarchical triads with (mean) prosociality score and densities of strong/weak links. We performed this analysis on the network and individual levels. On the individual level, for each student we calculated the density of balanced/hierarchical triads among ego-based triads this student is part of.

Tab. S7 shows that on the individual level, both densities (of hierarchical and balanced triads) are positively correlated with students' prosociality scores. The correlations are rather small but they are significant. Therefore, we can conclude that datasets contain information about the relation between prosociality and SBT or ST dynamics, but this information is not sufficient to differentiate how prosociality affects the competition between balance and status. The conclusions from our model suggest that with the increase of the prosociality, the group tends to favor SBT dynamics more.

The correlations between strong/weak positive/negative link densities with densities of balanced/hierarchical triads correspond to signs of edges. Positive edges are positively correlated with triad densities, and negative edges are anti-correlated with triad densities. This is not surprising. The result comes from the density of positive links influencing triad densities. In order to remove this influence, we performed a weighted linear regression analysis of the following models: $n_H \sim \rho + d_s$ and $n_B \sim \rho + d_s$, where ρ denotes the density of positive links, and d_s denotes the density of strong links (both positive and negative). In the analysis, weights were estimated in such a way that they are proportional to the inverse square of the variance. To be precise, to calculate weights (for instance, for $n_H \sim \rho + d_s$), first, we fitted simple linear regression and obtained model M_{ols}. Then, we

fitted simple linear regression with absolute residuals of M_{ols} as the response variable and fitted values of M_{ols} as the predictor variable. We obtained model M_w. Then, the weights are the inverse square of the fitted values of M_2.

Tab. S8 shows the analysis of the two models. The outcome is that the density of strong links does not have a significant influence on the density of hierarchical triads, but it does have a positive significant impact on the density of balanced triads. This conclusion matches the conclusion from fitting agent-based model parameters to dataset characteristics. With more strong relations, people are more likely to form balanced triads.

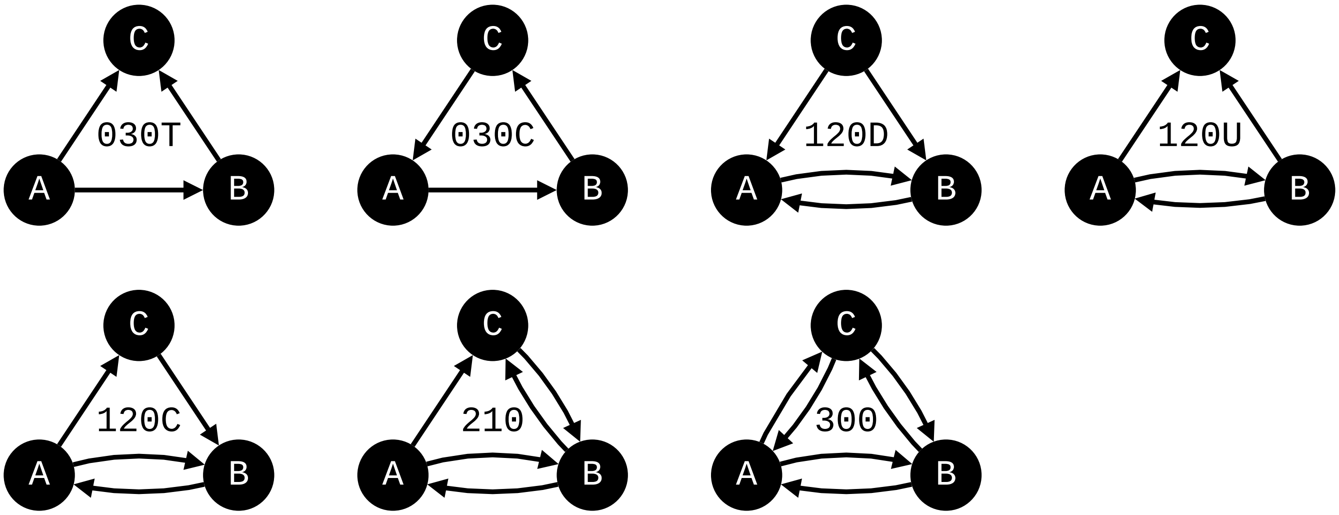

Fig. S1. Seven triadic motifs with connections existing between each pair of agents. Names of the motifs come from the triadic census. Only the motif 030C does not contain any ego-based triads. The rest are taken into account in the considered agent-based model dynamics.

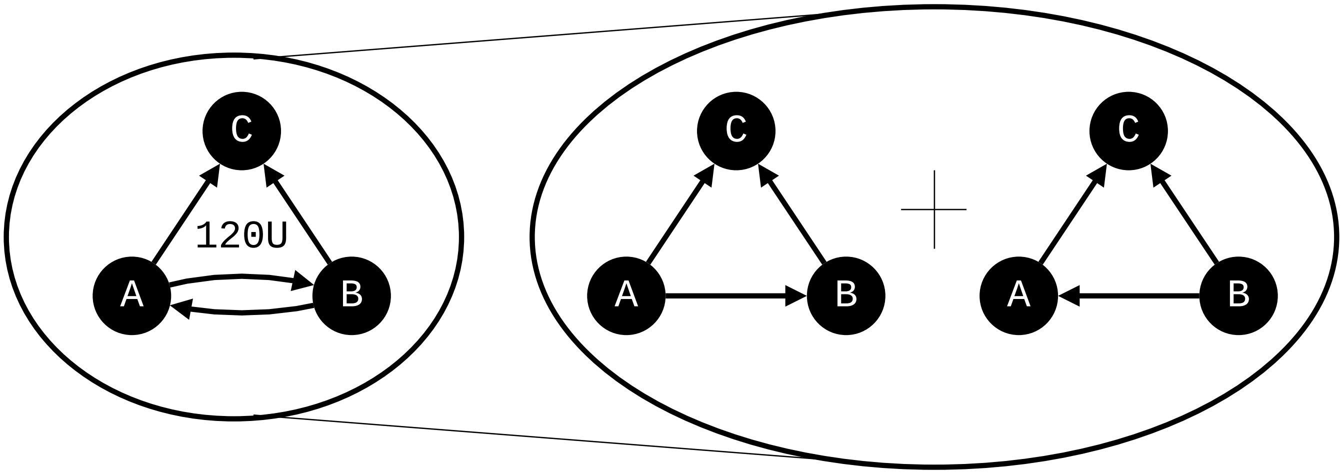

Fig. S2. Triadic motif 120U can be derived from two ego-based triads. In a trio of agents forming a 120U motif, not all links are considered within a single simulation step. However, in subsequent steps, different ego-based triads can be selected, ensuring that all links between the agents are eventually accounted for, just not at the same time.

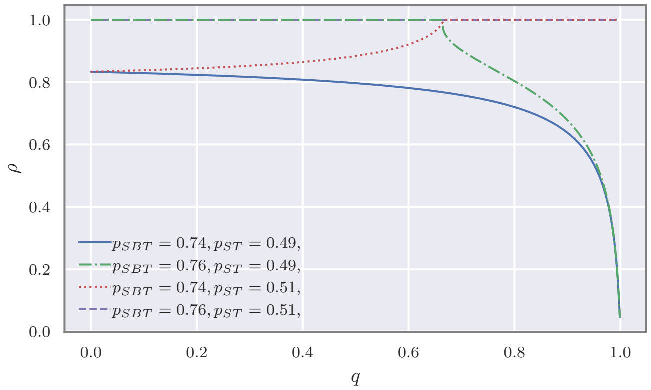

Fig. S3. Being above or below values $p_{SBT} = 0.75$ and $p_{ST} = 0.5$ is decisive for the transition type and the shape of the relation between positive link density ρ in equilibrium and the status importance q. For $p_{SBT} > 0.75$ and $p_{ST} < 0.5$, the transition is discontinuous (here, it is hardly visible because p_{SBT} and p_{ST} are very close to 0.75 and 0.5, respectively). For $p_{SBT} < 0.75$ and $p_{ST} > 0.5$, the transition is continuous. For other ranges of parameters, the transition is not observed. For $p_{SBT} > 0.75$ and $p_{ST} > 0.5$, the paradise state (i.e., $\rho = 1$) is the only solution for all values of q. For $p_{SBT} < 0.75$ and $p_{ST} < 0.5$, the unbalanced, quasi-stationary state exists for all values of q.

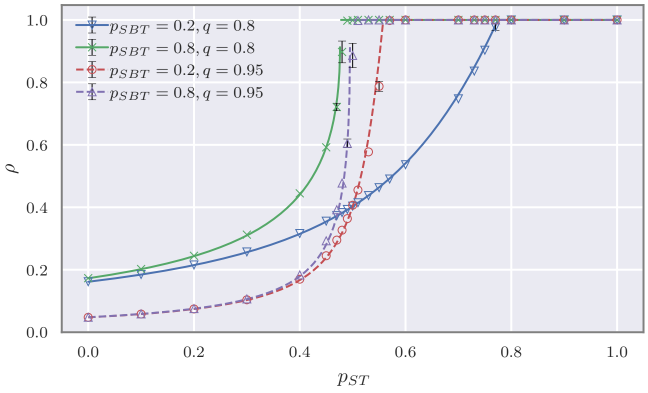

Fig. S4. Continuous and discontinuous phase transitions in density of positive links ρ as a function of parameter p_{ST}. For $p_{SBT} > 0.75$, transition is discontinuous. For $p_{SBT} < 0.75$, it is continuous. The lines indicate analytical results, and the markers are the results of agent-based simulations on a complete graph of 100 agents. Each simulation was repeated at least 100 times. Error bars representing standard deviations are shown when they exceed marker sizes.

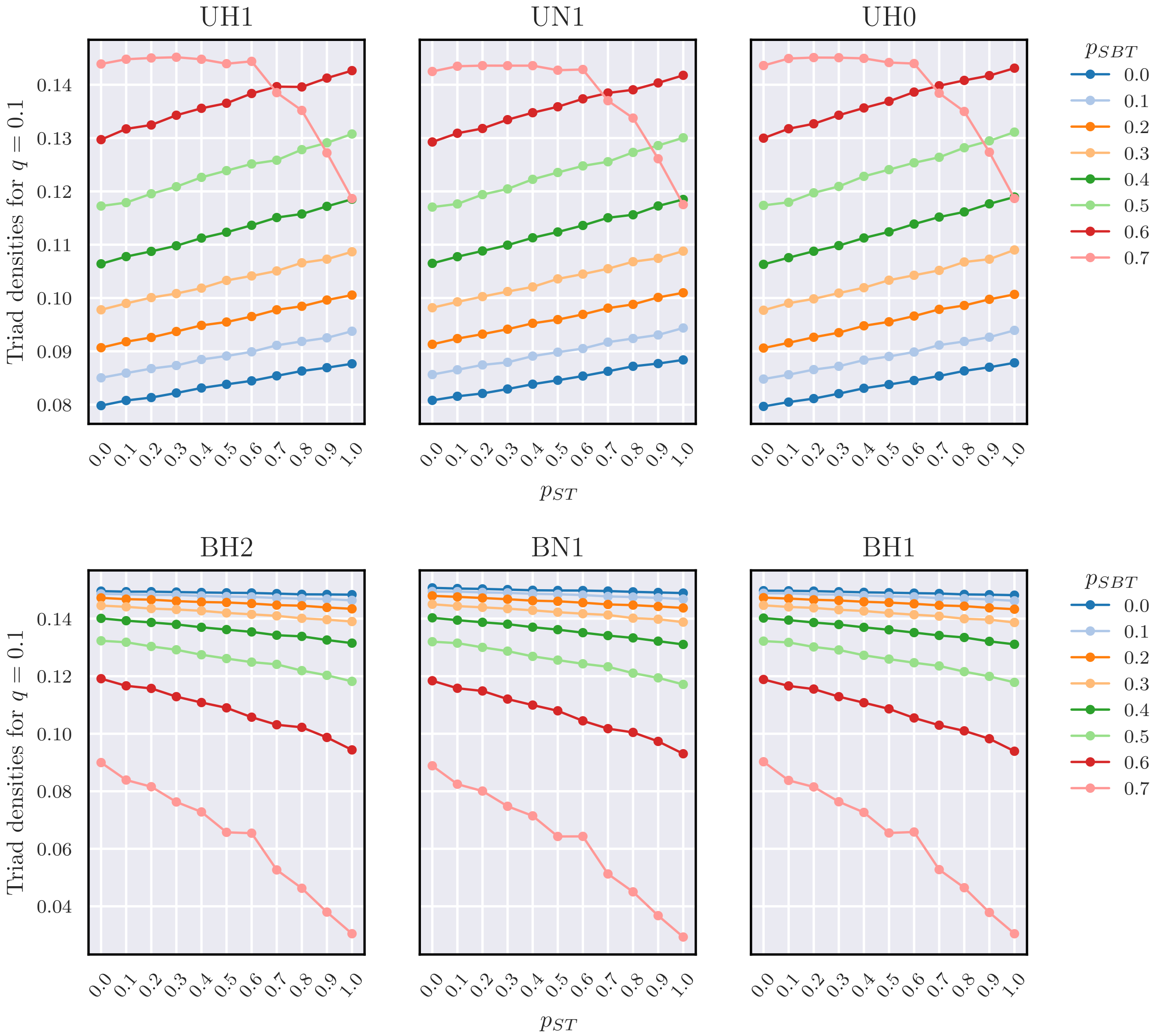

Fig. S5. Dependence of triad **densities** in the quasi-stationary state on preferences p_{SBT} and p_{ST} in the case of SBT being the dominant dynamics ($q = 0.1$). Plots show the results of agent-based simulations for a complete graph of 100 agents. The top or bottom rows represent unbalanced or balanced triads with 1 or 2 negative links, respectively. There is no visible difference of densities of different triads from the same group across different parameter values (p_{SBT}, p_{ST}). Each point represents a single simulation. Lines are a guide for the eyes.

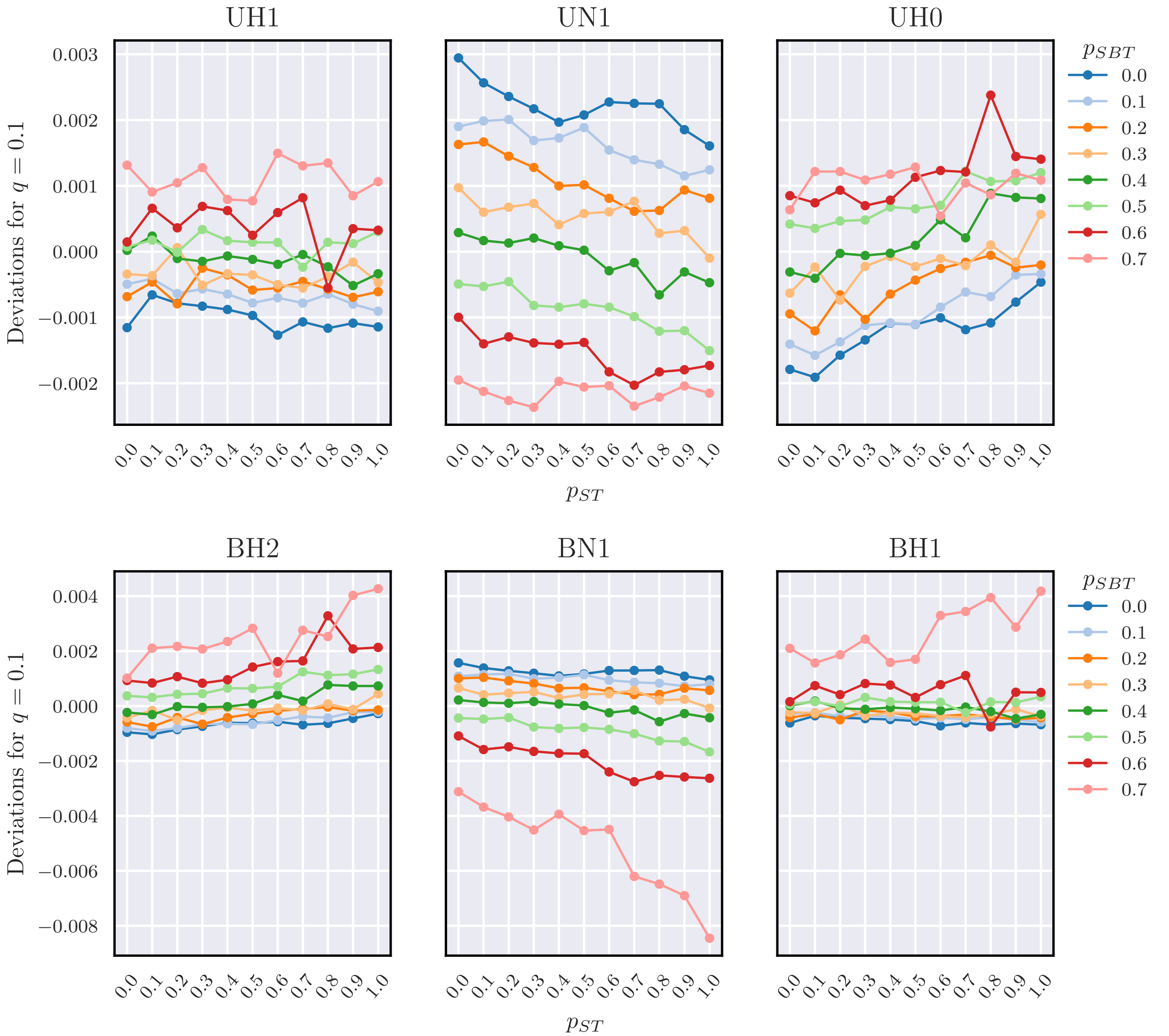

Fig. S6. Dependence of triad **deviations** in the quasi-stationary state on preferences p_{SBT} and p_{ST} in the case of SBT being the dominant dynamics ($q = 0.1$). Plots show results of agent-based simulations for a complete graph of 100 agents. The top or bottom rows represent unbalanced or balanced triads with 1 or 2 negative links, respectively. Small differences in densities of different triads from the same group are visible. In most cases, the change of p_{ST} does not influence the deviation. The increase of p_{SBT} leads to an increase of deviations for hierarchical triads and a decrease of deviations for nonhierarchical ones. Each point represents a single simulation. Lines are a guide for the eyes.

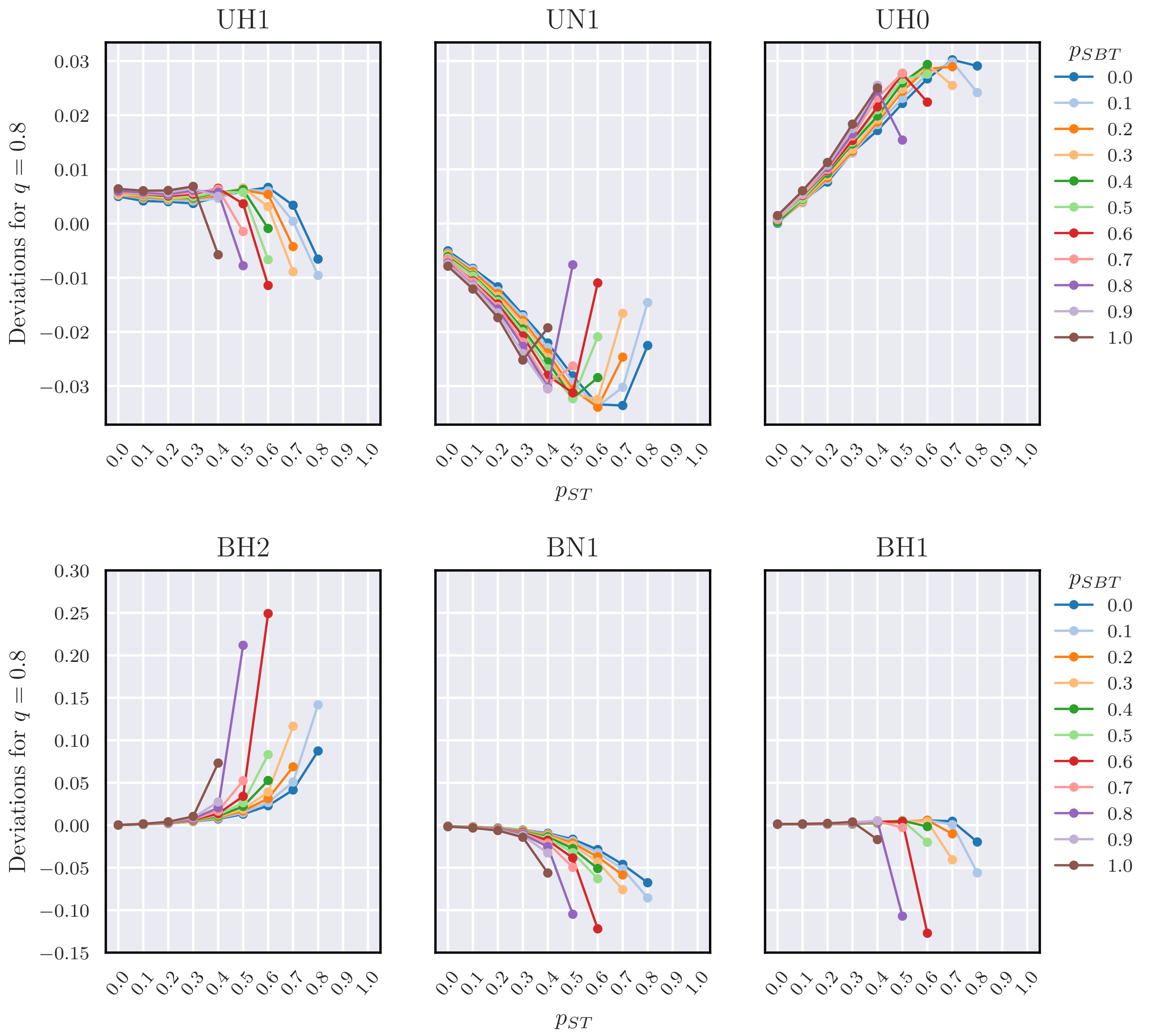

Fig. S7. Dependence of triad **deviations** in the quasi-stationary state on preferences p_{SBT} and p_{ST} in the case of ST being the dominant dynamics ($q = 0.8$). Plots show the results of agent-based simulations for a complete graph of 100 agents. The top or bottom rows represent unbalanced or balanced triads with 1 or 2 negative links, respectively. Large differences in densities of different triads within the same group are visible. In most cases, the increase of p_{ST} decreases the abundance of nonhierarchical triads and increases the abundance of hierarchical Δ_{UH0} and Δ_{BH2}. Deviations for triads Δ_{UH1} and Δ_{BH1} are usually close to 0, which means that the densities are around the expected levels. The change of p_{SBT} does not have much impact. Around the phase transition point, large and non-monotonic changes in deviation are observed. Each point represents at least 10 simulations. Error bars are not shown for better visibility. Lines are a guide for the eyes.

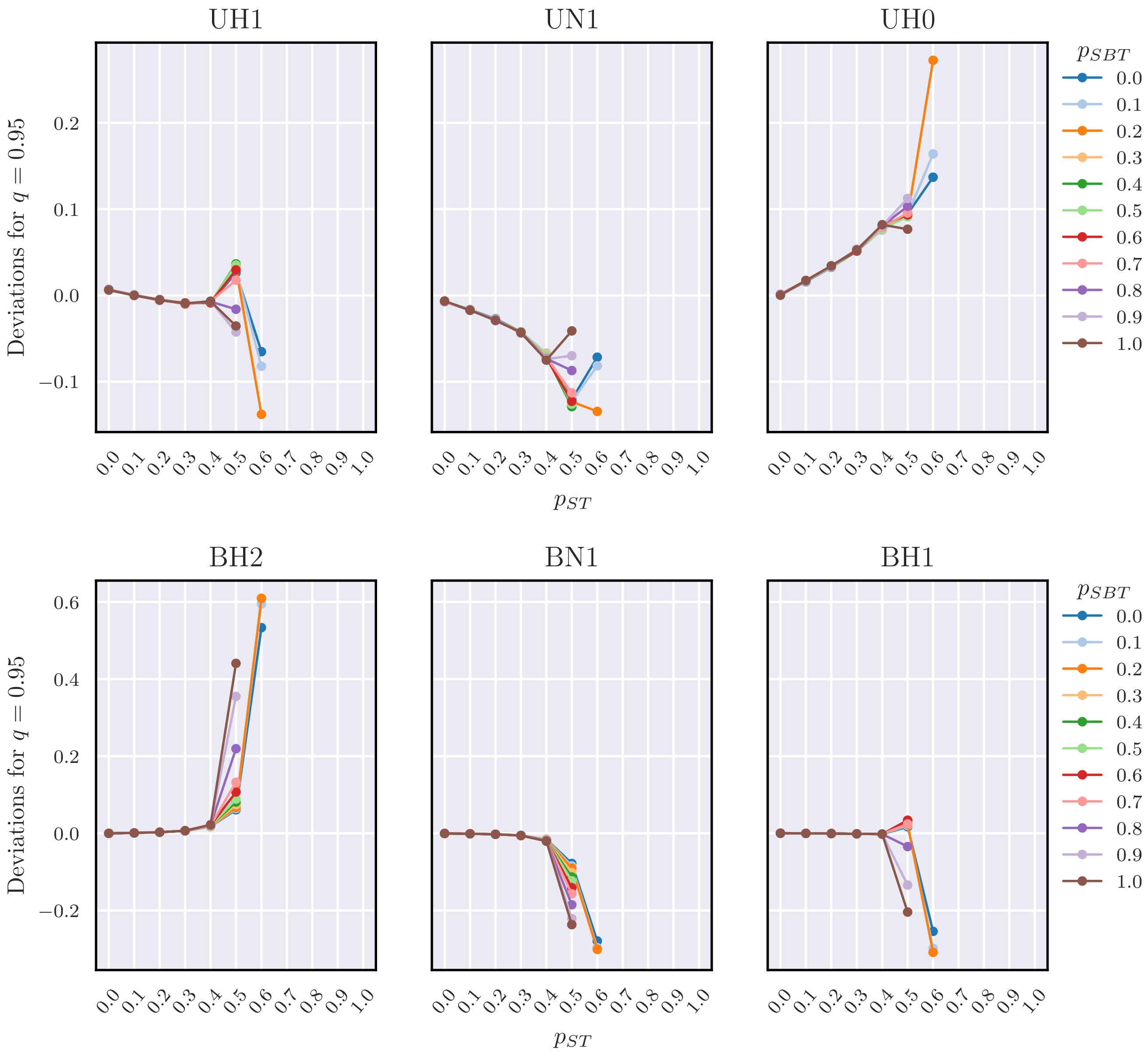

Fig. S8. Dependence of triad deviations in the quasi-stationary state on preferences p_{SBT} and p_{ST} in the case of ST being the **highly** dominant dynamics ($q = 0.95$). Plots show the results of agent-based simulations for a complete graph of 100 agents. The top or bottom rows represent unbalanced or balanced triads with 1 or 2 negative links, respectively. Comparing to Fig. S7, even larger differences in densities of different triads within the same group are visible. Deviations for triads Δ_{UH1} and Δ_{BH1} can be also positive. Each point represents at least 10 simulations. Error bars are not shown for better visibility. Lines are a guide for the eyes.

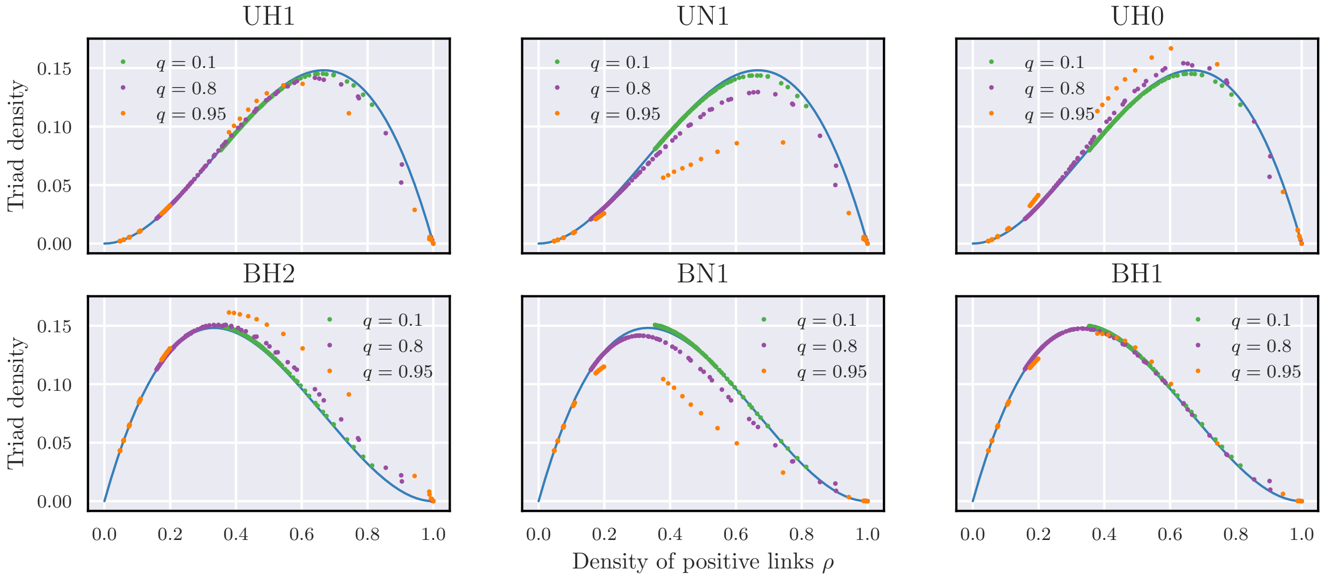

Fig. S9. Comparison of true triad densities to densities expected in the random network. Each data point represents a pair of densities (positive link density ρ and a quasi-stationary density of a given triad) that was obtained in agent-based simulations for different sets of parameters (q – coded with color, p_{SBT}, p_{ST}). The line represents the triad density in a random signed complete graph with a given density of positive links ρ. For $q = 0.1$, all triad densities are on the expected level. For larger values of q, only triads Δ_{BH1} and (approximately) Δ_{UH1} are correctly described by the random model. Hierarchical triads become over-represented, whereas nonhierarchical ones – under-represented.

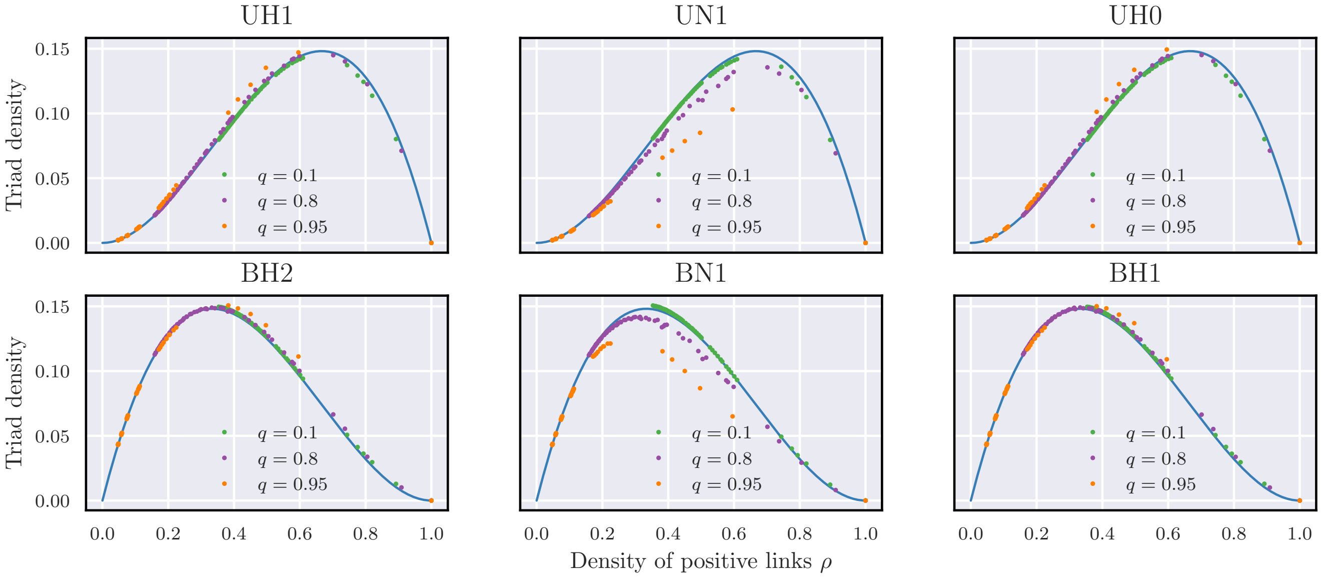

Fig. S10. Comparison of true triad densities to densities expected in the random network in the **triad-focused dynamics**. The densities of hierarchical triads are the same even for large q values. One could not recreate realistic deviations with triad-focused dynamics.

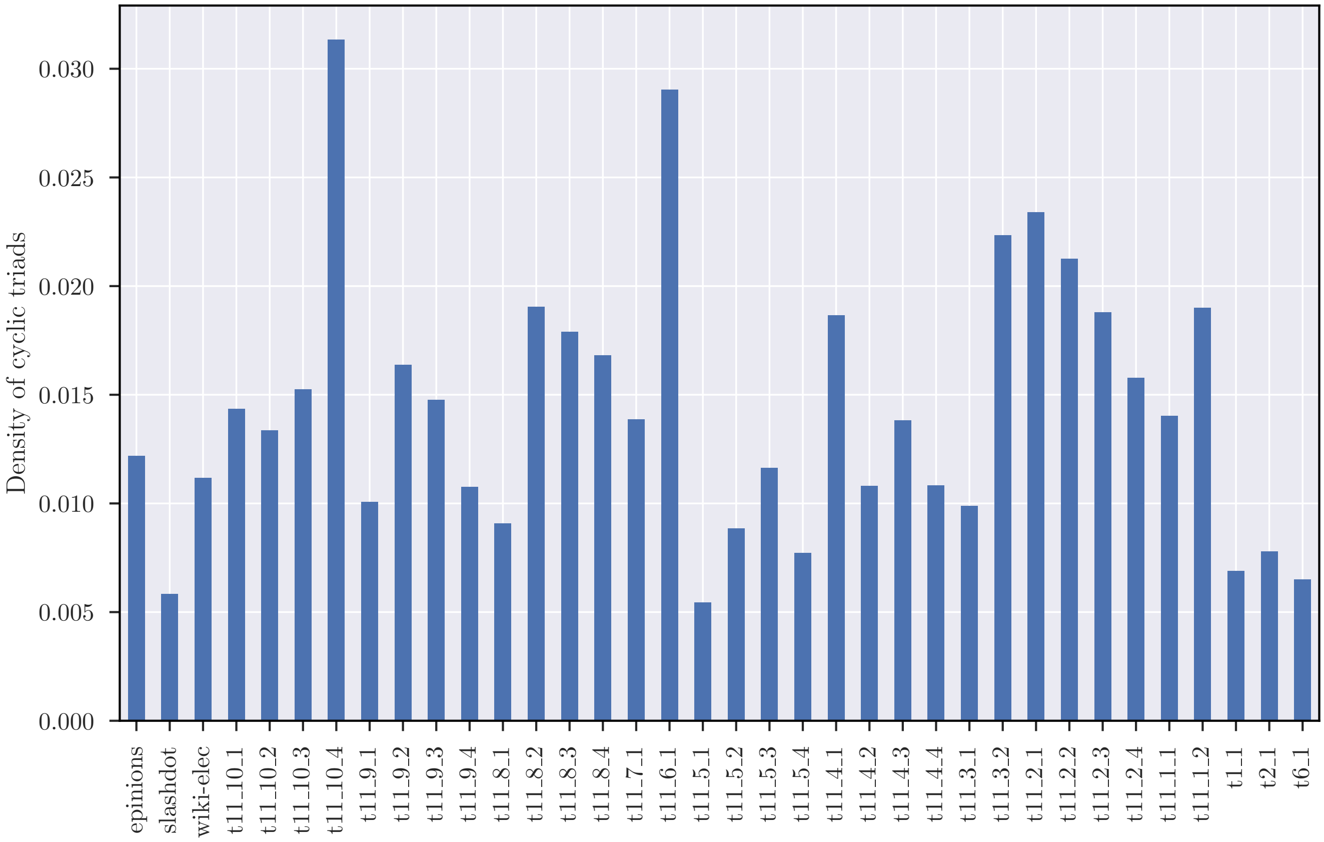

Fig. S11. Density of cyclic triads among all trios of agents with connections existing between each pair of agents for different datasets studied in this paper. Such trios of agents are not considered by the model dynamics. In terms of datasets analyzed, such motifs are a small fraction of all trios. The bars correspond to Epinions, Slashdot, WikiElections, and 33 networks from the high school dataset; see also the Data section from Materials and Methods from the main paper and section 4 from this document.

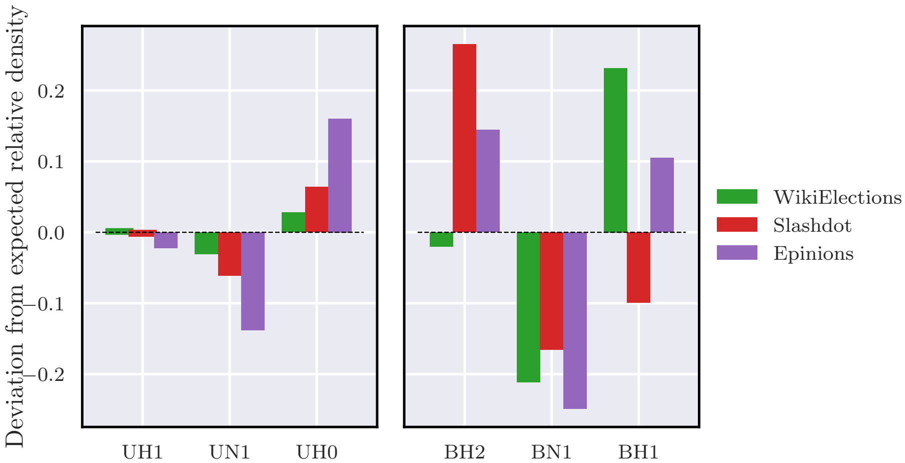

Fig. S12. Triad deviations for online networks.

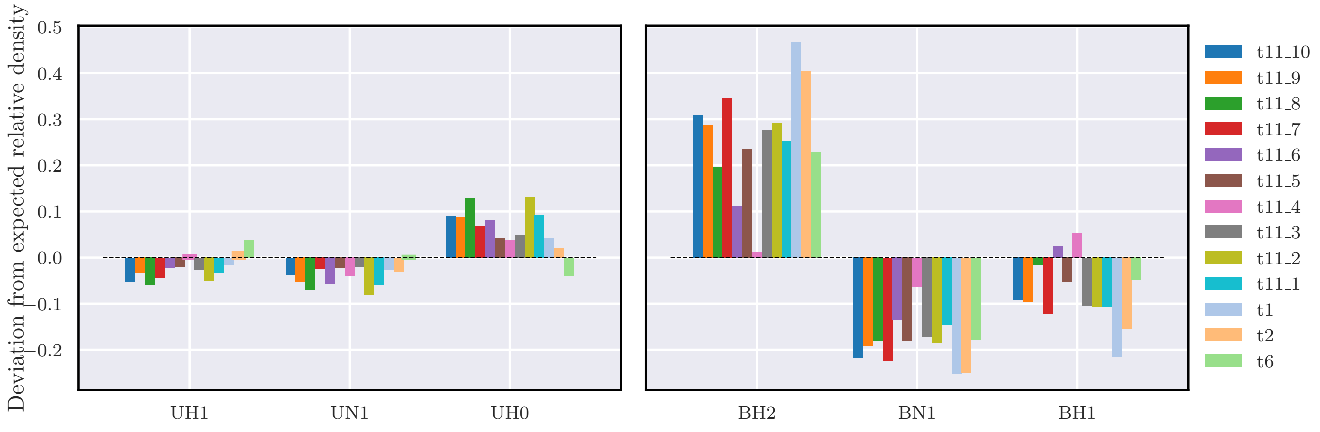

(a)

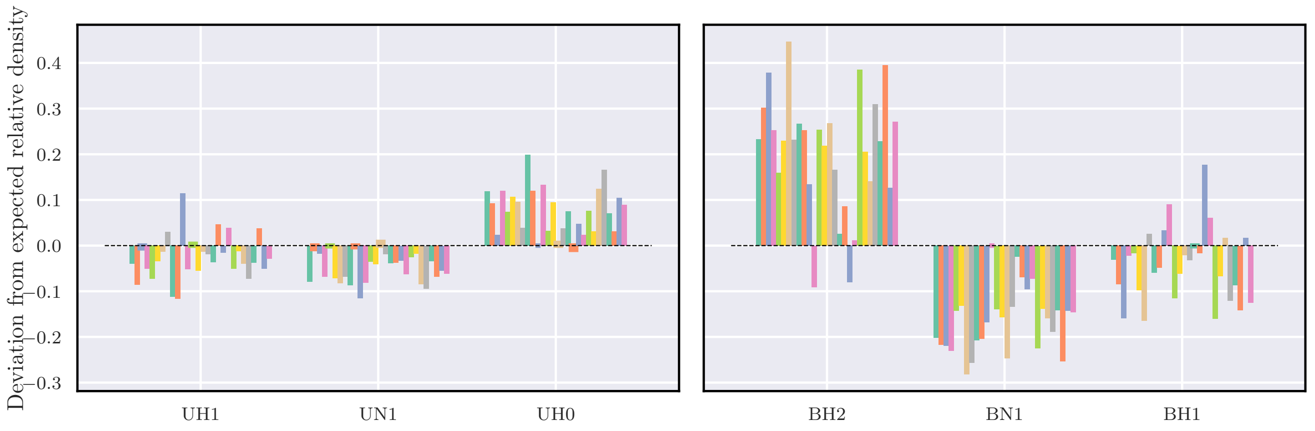

(b)

Fig. S13. Triad deviations for high school networks. Panel (a) shows average deviations for 8 schools (based on 28 networks) and exact deviations for 5 school-networks (t_11_7, t_11_6, $t1$, $t2$, $t6$). Panel (b) shows the exact deviations for those 28 networks. Almost in all cases, nonhierarchical triads are under-represented, and hierarchical triads (Δ_{UH0} and Δ_{BH2}) are over-represented.

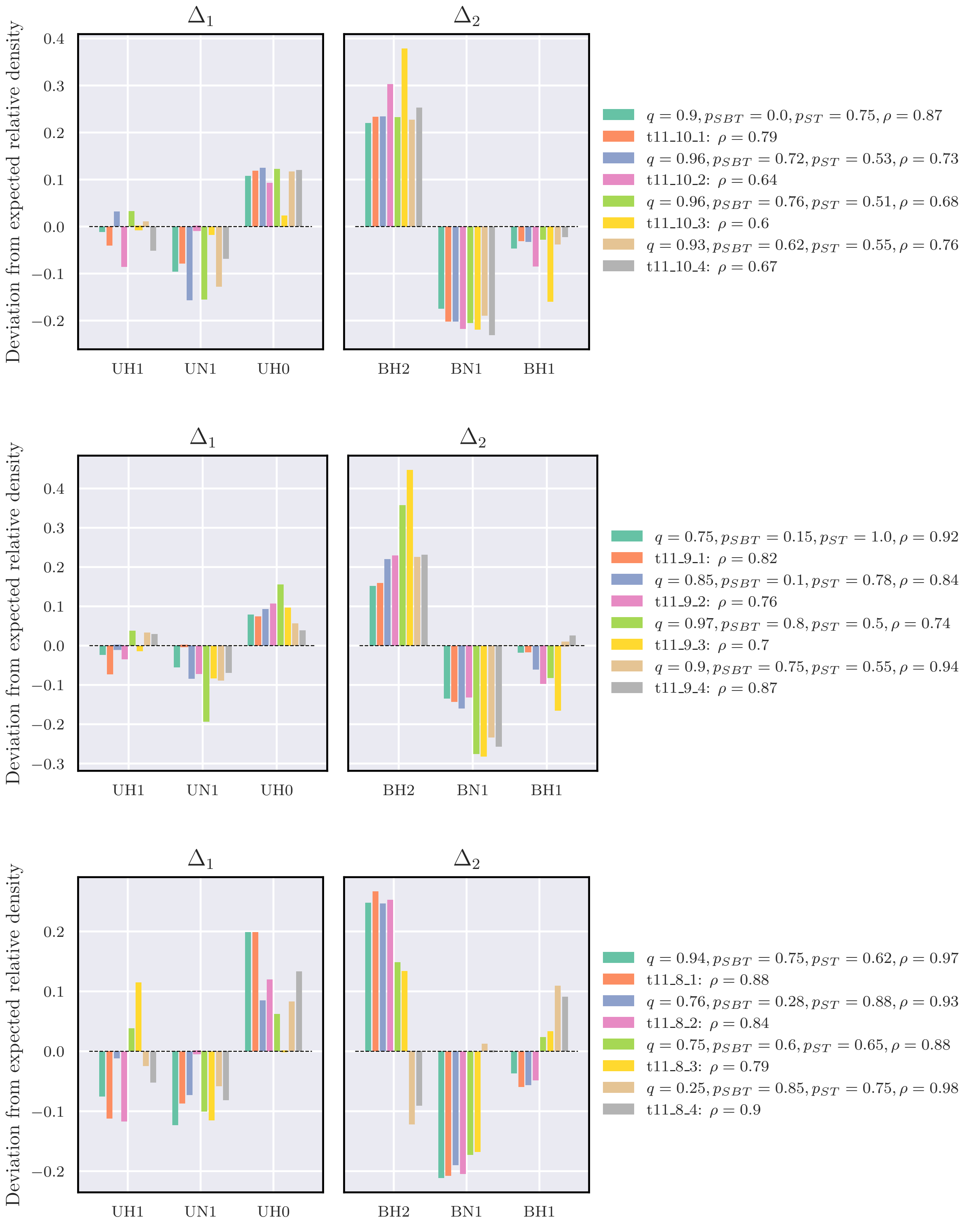

Fig. S14. Comparison between deviations from the best fit of agent-based model simulations and the true deviations for 12 high school networks. Each ABM deviations are followed by deviations for the respective school.

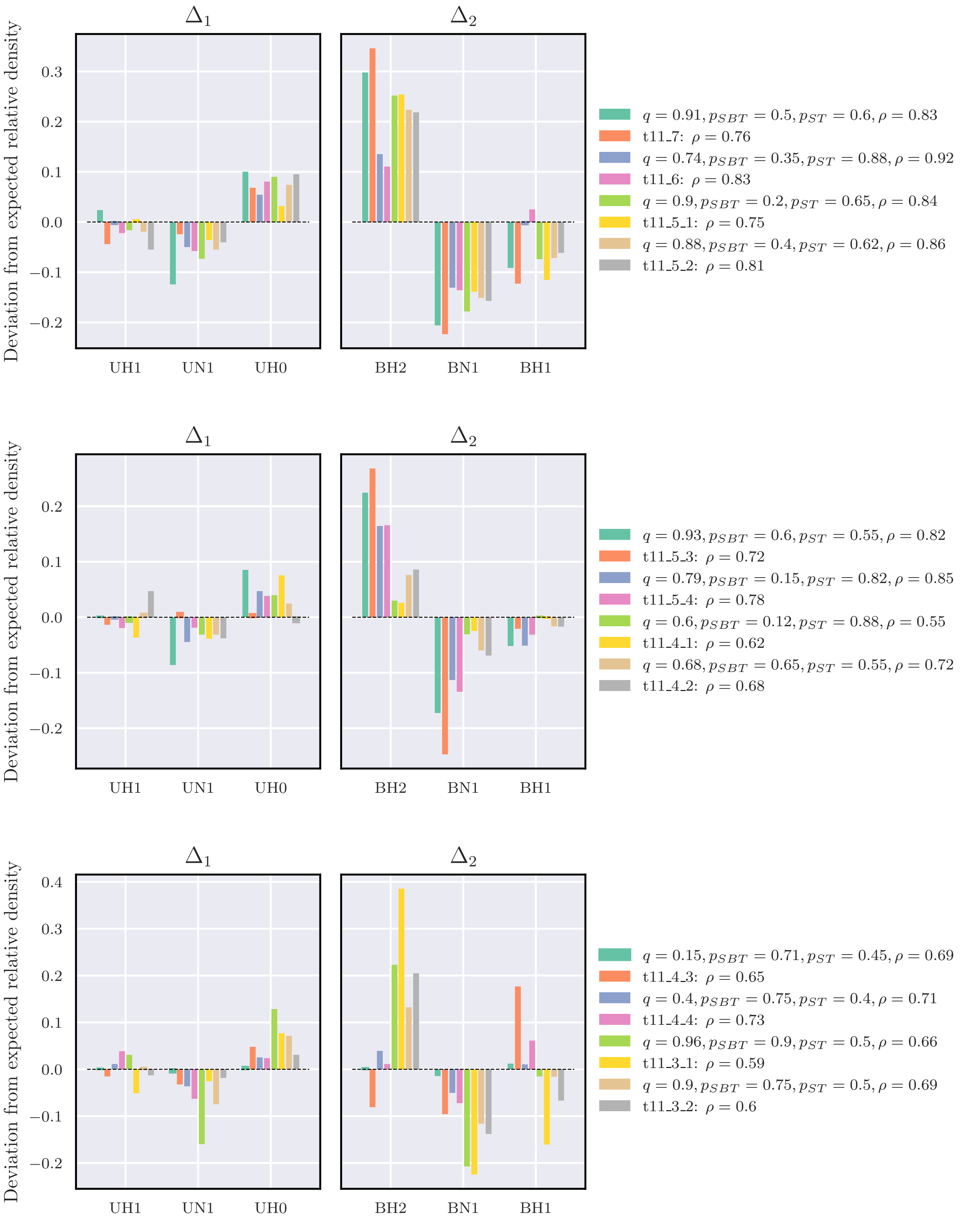

Fig. S15. Comparison between deviations from the best fit of agent-based model simulations and the true deviations for 12 high school networks. Each ABM deviations are followed by deviations for the respective school.

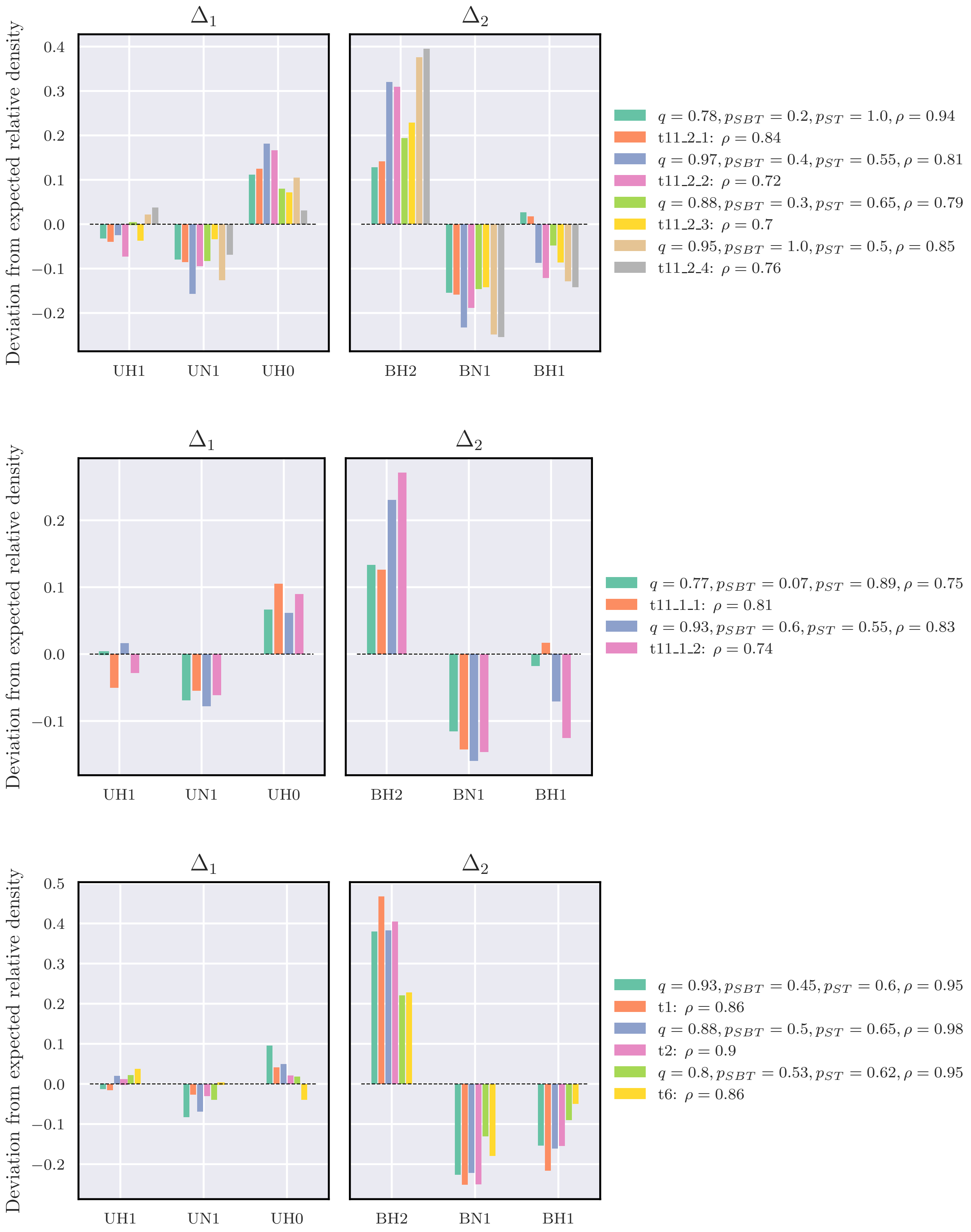

Fig. S16. Comparison between deviations from the best fit of agent-based model simulations and the true deviations for 9 high school networks. Each ABM deviations are followed by deviations for the respective school.

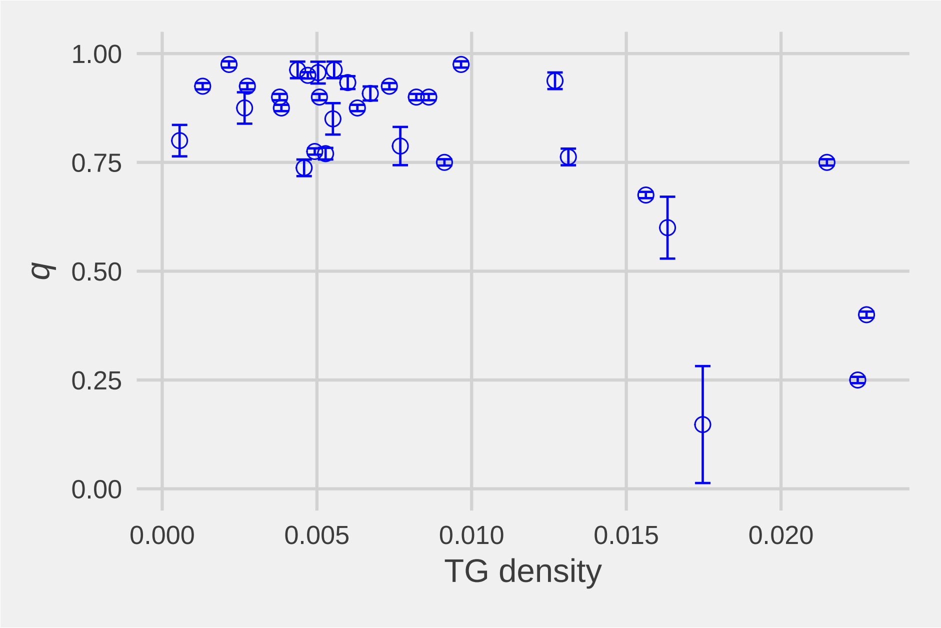

Fig. S17. Dependence of fitted values of status vs. structural balance importance q on the density in the triangle network for 33 high school networks.

Table S1. Number of pieces of external information required by a focal agent to evaluate a given triad. The triad names correspond to the triadic census motifs. These motifs, along with focal agents' labels, are shown in Fig. S1. The table shows that agent A of the motif 030T requires the least amount of external information.

Triad	Focal agent	Pieces of external information
030T	A	1
030T	B	2
030T	C	3
030C	(any)	2
120D	A	3
120D	B	3
120D	C	2
120U	A	2
120U	B	2
120U	C	4
120C	A	2
120C	B	3
120C	C	3
210	A	3
210	B	3
210	C	4
300	(any)	4

Table S2. Properties of online networks. Considered triads are motifs consisting of three agents and three links not forming loops (see Fig. 1 in the main paper). Among each trio of agents, there can be 6 triads.

	WikiElections	Slashdot	Epinions
Number of agents	7,118	82,140	131,828
Number of links	103,675	549,202	841,372
Positive link density	0.784	0.774	0.853
Number of links in triads	94,933	285,003	669,890
Positive link density in triads	0.795	0.777	0.874
Number of triads	745,129	1,251,925	11,005,985
Number of agents in triads	3,973	26,725	43,862
Number of focal agents	2,816	16,438	30,727

Table S3. Network properties of high school dataset. Column *Network* contains a coded school name extended with students' grades. ρ denotes positive link density after removing edges not in triads (see text for details). The last two columns describe the properties of constructed triangle networks.

Network	Agents	Focal agents	Edges	ρ	Triads	Density	Edges in TG	TG density
t11_10_1	94	90	1,486	0.787	9,598	0.170	468,005	0.00508
t11_10_2	98	95	1,760	0.644	14,186	0.185	880,634	0.00438
t11_10_3	99	96	1,545	0.600	9,564	0.159	460,677	0.00504
t11_10_4	95	91	1,325	0.669	7,494	0.148	336,743	0.00600
t11_9_1	67	64	838	0.822	5,246	0.190	250,995	0.00912
t11_9_2	94	92	1,309	0.756	7,569	0.150	316,034	0.00552
t11_9_3	75	68	782	0.696	3,175	0.141	97,343	0.00966
t11_9_4	82	74	934	0.875	4,240	0.141	147,601	0.00821
t11_8_1	52	48	568	0.880	3,084	0.214	120,707	0.01270
t11_8_2	67	64	599	0.840	2,213	0.135	64,256	0.01313
t11_8_3	47	42	356	0.787	1,156	0.165	28,684	0.02148
t11_8_4	41	37	308	0.903	1,015	0.188	23,136	0.02248
t11_7	80	75	1,040	0.759	5,704	0.165	218,789	0.00673
t11_6	106	103	1,637	0.827	9,363	0.147	402,036	0.00459
t11_5_1	52	52	799	0.750	7,527	0.301	487,995	0.00861
t11_5_2	59	59	1,093	0.810	11,108	0.319	778,183	0.00631
t11_5_3	56	55	957	0.723	9,451	0.311	655,665	0.00734
t11_5_4	56	56	918	0.782	8,737	0.298	587,303	0.00769
t11_4_1	29	28	394	0.617	3,212	0.485	168,443	0.01633
t11_4_2	27	27	396	0.684	3,798	0.564	225,423	0.01563
t11_4_3	28	28	377	0.650	3,172	0.499	175,718	0.01747
t11_4_4	26	26	269	0.732	1,376	0.414	43,069	0.02276
t11_3_1	73	70	1,304	0.594	12,077	0.248	810,371	0.00556
t11_3_2	83	81	1,797	0.600	19,904	0.264	1,502,710	0.00379
t11_2_1	114	110	1,554	0.845	7,735	0.121	294,905	0.00493
t11_2_2	155	150	3,696	0.719	34,789	0.155	2,612,608	0.00216
t11_2_3	118	114	1,923	0.696	12,537	0.139	605,298	0.00385
t11_2_4	125	114	1,736	0.757	10,103	0.112	480,210	0.00471
t11_1_1	107	99	1,413	0.808	7,551	0.125	301,058	0.00528
t11_1_2	125	121	2,773	0.736	26,585	0.179	1,944,631	0.00275
t1	326	290	5,894	0.859	45,760	0.056	2,738,462	0.00131
t2	202	175	3,011	0.902	24,365	0.074	1,580,672	0.00266
t6	534	483	12,777	0.860	143,112	0.045	11,503,867	0.00056
SUM	3,392	3,177	57,568		476,506			

Table S4. Students' and students' relations' properties in high school dataset. Column *Network* contains a coded school name extended with students' grades. *Link densities* sum up to 1. *Boys* column represents the ratio of boys. For details about *Prosociality* score and Cognitive Reflection Test (*CRT*) score, see (5).

Network	Classes	Link densities				Boys	Prosociality score		CRT score	
		Strong negative	Weak negative	Weak positive	Strong positive		Mean	Std	Mean	Std
t11_10_1	4	0.061	0.152	0.504	0.283	0.479	0.643	0.270	1.309	0.892
t11_10_2	4	0.054	0.302	0.440	0.204	0.500	0.641	0.283	1.378	0.767
t11_10_3	4	0.044	0.356	0.408	0.192	0.566	0.614	0.274	1.727	0.740
t11_10_4	4	0.040	0.291	0.451	0.218	0.537	0.605	0.286	1.737	0.828
t11_9_1	4	0.044	0.134	0.579	0.243	0.507	0.588	0.297	1.507	0.704
t11_9_2	5	0.057	0.188	0.509	0.247	0.500	0.650	0.257	1.479	0.699
t11_9_3	5	0.054	0.251	0.439	0.257	0.453	0.584	0.306	1.587	0.755
t11_9_4	4	0.016	0.109	0.615	0.260	0.537	0.684	0.240	1.732	0.649
t11_8_1	3	0.058	0.062	0.634	0.246	0.385	0.706	0.270	1.192	0.841
t11_8_2	5	0.038	0.122	0.529	0.311	0.567	0.563	0.293	1.313	0.679
t11_8_3	3	0.051	0.163	0.528	0.258	0.362	0.660	0.256	1.511	0.655
t11_8_4	3	0.026	0.071	0.594	0.308	0.439	0.717	0.253	1.366	0.915
t11_7	4	0.084	0.158	0.473	0.286	0.563	0.689	0.240	1.050	0.745
t11_6	5	0.064	0.109	0.539	0.288	0.509	0.569	0.339	1.226	0.808
t11_5_1	2	0.075	0.175	0.528	0.222	0.481	0.635	0.326	1.404	0.846
t11_5_2	2	0.038	0.153	0.530	0.280	0.373	0.679	0.224	1.424	0.835
t11_5_3	2	0.098	0.179	0.470	0.253	0.518	0.692	0.262	1.786	0.706
t11_5_4	2	0.053	0.164	0.485	0.297	0.571	0.674	0.243	1.821	0.897
t11_4_1	1	0.147	0.236	0.345	0.272	0.517	0.563	0.336	1.207	0.861
t11_4_2	1	0.131	0.184	0.391	0.293	0.481	0.730	0.262	1.407	0.636
t11_4_3	1	0.061	0.289	0.310	0.340	0.500	0.703	0.278	1.500	0.882
t11_4_4	1	0.052	0.216	0.446	0.286	0.500	0.591	0.304	1.808	1.021
t11_3_1	3	0.094	0.311	0.333	0.262	0.603	0.636	0.325	1.575	0.575
t11_3_2	3	0.126	0.274	0.319	0.281	0.446	0.660	0.245	1.663	0.720
t11_2_1	6	0.035	0.120	0.543	0.302	0.491	0.650	0.288	1.263	0.776
t11_2_2	7	0.048	0.233	0.514	0.205	0.542	0.707	0.247	1.561	0.748
t11_2_3	6	0.068	0.237	0.492	0.204	0.542	0.631	0.292	1.686	0.736
t11_2_4	6	0.039	0.204	0.505	0.252	0.480	0.654	0.246	1.592	0.862
t11_1_1	5	0.050	0.142	0.577	0.231	0.467	0.677	0.285	1.290	0.869
t11_1_2	5	0.064	0.200	0.533	0.203	0.464	0.663	0.255	1.448	0.788
t1	21	0.036	0.105	0.580	0.279	0.531	0.744	0.240	2.224	0.746
t2	10	0.046	0.052	0.528	0.374	0.520	0.761	0.252	1.554	0.919
t6	18	0.047	0.092	0.482	0.378	0.528	0.800	0.244	2.150	0.995

Table S5. Fitted values of parameters and their uncertainties for different schools.

School	q	p_{SBT}	p_{ST}	δ_q	$\delta_{p_{SBT}}$	$\delta_{p_{ST}}$
t11_10_1	0.900	0.000	0.750	0.0072	0.014	0.014
t11_10_2	0.963	0.725	0.525	0.019	0.319	0.038
t11_10_3	0.956	0.763	0.513	0.025	0.218	0.029
t11_10_4	0.933	0.625	0.550	0.015	0.305	0.057
t11_9_1	0.750	0.150	1.000	0.0072	0.014	0.014
t11_9_2	0.850	0.100	0.775	0.036	0.072	0.038
t11_9_3	0.975	0.800	0.500	0.0072	0.014	0.014
t11_9_4	0.900	0.750	0.550	0.0072	0.014	0.014
t11_8_1	0.938	0.750	0.625	0.019	0.072	0.038
t11_8_2	0.763	0.275	0.875	0.019	0.107	0.107
t11_8_3	0.750	0.600	0.650	0.0072	0.014	0.014
t11_8_4	0.250	0.850	0.750	0.0072	0.014	0.014
t11_7	0.908	0.500	0.600	0.016	0.427	0.101
t11_6	0.738	0.350	0.875	0.019	0.072	0.038
t11_5_1	0.900	0.200	0.650	0.0072	0.014	0.014
t11_5_2	0.875	0.400	0.625	0.0072	0.354	0.107
t11_5_3	0.925	0.600	0.550	0.0072	0.014	0.014
t11_5_4	0.788	0.150	0.825	0.044	0.235	0.156
t11_4_1	0.600	0.125	0.875	0.071	0.107	0.177
t11_4_2	0.675	0.650	0.550	0.0072	0.014	0.014
t11_4_3	0.148	0.710	0.445	0.134	0.078	0.307
t11_4_4	0.400	0.750	0.400	0.0072	0.014	0.014
t11_3_1	0.963	0.900	0.500	0.019	0.072	0.014
t11_3_2	0.900	0.750	0.500	0.0072	0.014	0.014
t11_2_1	0.775	0.200	1.000	0.0072	0.014	0.014
t11_2_2	0.975	0.400	0.550	0.0072	0.014	0.014
t11_2_3	0.875	0.300	0.650	0.0072	0.014	0.014
t11_2_4	0.950	1.000	0.500	0.0072	0.014	0.014
t11_1_1	0.770	0.070	0.890	0.013	0.099	0.076
t11_1_2	0.925	0.600	0.550	0.0072	0.014	0.014
t1	0.925	0.450	0.600	0.0072	0.014	0.014
t2	0.875	0.500	0.650	0.036	0.495	0.213
t6	0.800	0.525	0.625	0.036	0.107	0.038

Table S6. Description of significant linear regression models. Four models of two or three exploratory variables are given. Columns present model variables, model test p-values, variable coefficients (with p-values indicated), coefficients' standard errors (SE), R-squared, and adjusted R-squared metrics. Densites of weakly negative, weakly positive and strongly positive are denoted by d_{w-}, d_{w+} and d_{s+}, respectively. Mean prosociality, standard deviation of prosociality and density of students with lowest prosociality score are denoted by $\overline{\text{prosoc}}$, std prosoc and soc_0, respectively. Density of triads with two negative links is denoted by n_2.

Model variables			Model p-value	Variable coefficients			SE of coefficients			R^2	Adjusted R^2
1	2	3		1	2	3	1	2	3		
d_{w-}	$\overline{\text{prosoc}}$		1.99×10^{-4}	0.76***	0.80**		0.17	0.23		0.51	0.47
n_2	soc_0		1.37×10^{-3}	0.48***	-0.66*		0.13	0.26		0.42	0.37
d_{s+}	std prosoc		3.46×10^{-2}	-0.81*	-0.87*		0.36	0.41		0.24	0.18
n_2	std prosoc	d_{w+}	1.52×10^{-4}	1.19***	-1.02**	0.95**	0.27	0.31	0.31	0.58	0.52

Significance p-value codes: $p < 0.001$ ***, $p < 0.01$ **, $p < 0.05$ *

Table S7. Pearson correlations between prosociality score with densities of hierarchical n_H and structurally balanced n_B triads, and between densities of strong/weak positive/negative links with densities n_H and n_B. For the prosociality score, on the network level, the correlation was calculated using average values.

Correlation between	Network level	Individual level
prosociality and n_H	0.25	0.10 (***)
prosociality and n_B	0.35 (*)	0.14 (***)
strong pos density and n_H	0.07	0.31 (***)
weak pos density and n_H	0.77 (***)	0.40 (***)
weak neg density and n_H	$-$0.54 (**)	$-$0.52 (***)
strong neg density and n_H	$-$0.78 (***)	$-$0.49 (***)
weak neg density and n_B	0.37 (*)	0.44 (***)
weak pos density and n_B	0.65 (***)	0.42 (***)
weak neg density and n_B	$-$0.67 (***)	$-$0.66 (***)
strong neg density and n_B	$-$0.64 (***)	$-$0.49 (***)

Significance p-value codes: $p < 0.001$ ***, $p < 0.01$ **, $p < 0.05$ *

Table S8. Parameters of weighted linear regression analysis of models showing the influence of density of strong links d_s on densities of hierarchical n_H and balanced n_B triads while controlling the density of positive links ρ. The density of strong links does not explain the density of hierarchical triads, but it does explain the density of balanced triads.

Model	Model p-value	Coefficients		SE of coefficients		R^2	Adjusted R^2
		ρ	d_s	ρ	d_s		
$n_H \sim \rho + d_s$	***	0.2231***	-0.0015	0.0045	0.0039	0.43	0.42
$n_B \sim \rho + d_s$	***	0.5345***	0.0533***	0.0088	0.0087	0.54	0.54

Significance p-value codes: $p < 0.001$ ***, $p < 0.01$ **, $p < 0.05$ *

References

1. P Holland, S Leinhardt, *The Statistical Analysis of Local Structure in Social Networks.* (1974).
2. T Antal, PL Krapivsky, S Redner, Dynamics of social balance on networks. *Phys. Rev. E* **72**, 36121 (2005).
3. [dataset] Ryan A. Rossi, NK Ahmed, The network data repository with interactive graph analytics and visualization in *Proceedings of the Twenty-Ninth AAAI Conference on Artificial Intelligence.* (2015).
4. [dataset] Miguel Ruíz-García, et al., Triadic influence as a proxy for compatibility in social relationships (https://doi.org/10.5281/zenodo.7647000) (2023).
5. M Ruiz-García, et al., Triadic influence as a proxy for compatibility in social relationships. *Proc. Natl. Acad. Sci. United States Am.* **120**, e2215041120 (2023).
